# Nanozyme as a rising star for metabolic disease management

**DOI:** 10.1186/s12951-024-02478-5

**Published:** 2024-05-06

**Authors:** Yanan Wang, Xiaoyun He, Kunlun Huang, Nan Cheng

**Affiliations:** 1 Beijing Laboratory for Food Quality and Safety, College of Food Science and Nutritional Engineering, China Agricultural University, No. 17 Qinghua East Road, Haidian District, Beijing, 100083 People’s Republic of China; 2 Key Laboratory of Safety Assessment of Genetically Modified Organism (Food Safety), The Ministry of Agriculture and Rural Affairs of the PR China, Beijing, China

**Keywords:** Nanozyme, Metabolic disease, Inflammation, Oxidative stress, Glucose homeostasis

## Abstract

Nanozyme, characterized by outstanding and inherent enzyme-mimicking properties, have emerged as highly promising alternatives to natural enzymes owning to their exceptional attributes such as regulation of oxidative stress, convenient storage, adjustable catalytic activities, remarkable stability, and effortless scalability for large-scale production. Given the potent regulatory function of nanozymes on oxidative stress and coupled with the fact that reactive oxygen species (ROS) play a vital role in the occurrence and exacerbation of metabolic diseases, nanozyme offer a unique perspective for therapy through multifunctional activities, achieving essential results in the treatment of metabolic diseases by directly scavenging excess ROS or regulating pathologically related molecules. The rational design strategies, nanozyme-enabled therapeutic mechanisms at the cellular level, and the therapies of nanozyme for several typical metabolic diseases and underlying mechanisms are discussed, mainly including obesity, diabetes, cardiovascular disease, diabetic wound healing, and others. Finally, the pharmacokinetics, safety analysis, challenges, and outlooks for the application of nanozyme are also presented. This review will provide some instructive perspectives on nanozyme and promote the development of enzyme-mimicking strategies in metabolic disease therapy.

**Graphical Abstract:**

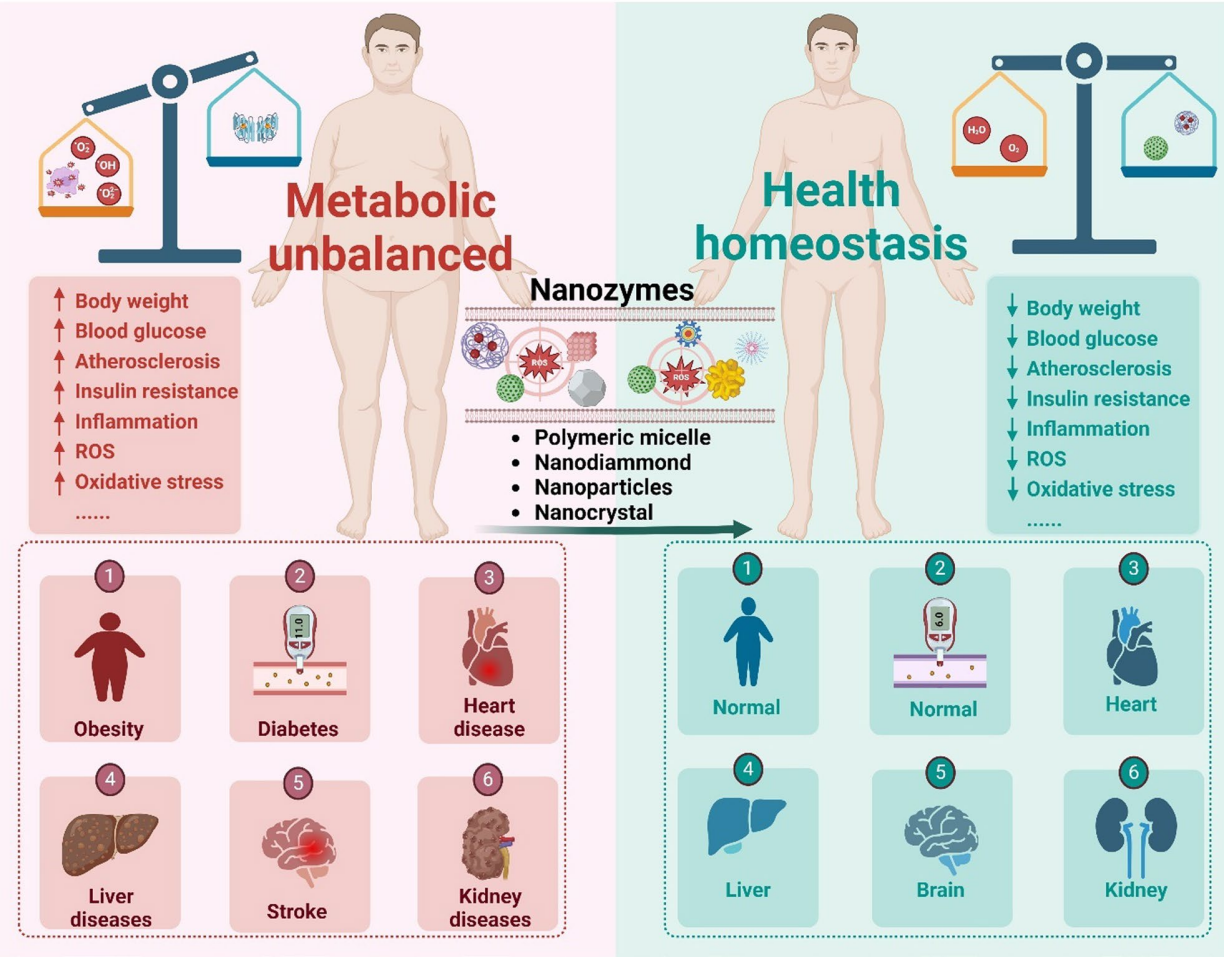

## Introduction

Nanozymes, possessing unique properties of nanomaterials and catalytic functions, are characterized by high catalytic efficiency, stability, cost-effectiveness, and scalability, and find widespread applications in medicine, chemical engineering, food, agriculture, and the environment [1–3]. The concept of a “nanozyme” was first proposed in 2004 and was originally referred to nanoparticle-based enzyme mimics [4]. Until 2007, researchers discovered that Fe_3_O_4_ nanoparticles exhibit peroxidase (POD)-like activity [5]. Since then, the traditional notion of inorganic materials being biologically inert has been shattered, leading to widespread interest among researchers in exploring other novel enzyme-like nanomaterials. With the development of nanotechnology, numerous studies have shown that nanozymes have the function of regulating reactive oxygen species (ROS). Based on this, nanozymes are widely used in the regulation and improvement of a variety of ROS-related diseases [6–8].

Metabolic diseases, such as obesity, diabetes, and cardiovascular disorders, have emerged as major global health concerns, posing significant challenges to global healthcare systems [9–11]. These diseases are characterized by impaired metabolic processes, such as increased body weight, blood glucose, insulin resistance, inflammation, and oxidative stress, leading to detrimental effects on overall health and quality of life [12–15]. Notably, oxidative stress is a key factor in the onset and progression of metabolic diseases [16]. The overabundance of free radicals leads to the functional impairment of proteins, lipids, and DNA in cells and tissues, ultimately disrupting normal signaling pathways and inflicting harm on the body [17]. Approximately 2 billion adults worldwide are overweight, and around 650 million individuals suffer from obesity, as reported by the World Health Organization (WHO) [18]. In addition, data from the International Diabetes Federation suggests that in 2019, an estimated 463 million adults were diagnosed with diabetes globally. This number is projected to escalate to approximately 700 million individuals with diabetes over the next 25 years [19]. The alarming numbers serve as a wake-up call for the immediate implementation of measures to prevent and treat metabolic disorders.

Traditional approaches to metabolic disease management, such as pharmacological interventions and lifestyle modifications, have limitations in terms of safety and efficacy [20–22]. Many medications used to manage metabolic diseases can have significant side effects. For example, metformin commonly causes side effects such as diarrhea, nausea, vomiting, flatulence, abdominal pain, loss of appetite, and lactic acidosis [23–25]. Over time, patients may develop tolerance to medications, necessitating higher doses or alternative treatments, while certain metabolic diseases may also exhibit resistance to specific medications. Furthermore, individuals with metabolic diseases often necessitate multiple medications and long-term pharmacological interventions, thereby heightening the likelihood of drug interactions and adverse effects. Therefore, there is a pressing need for innovative strategies that can improve disease management. Recent advancements in nanotechnology, specifically in the development of nanozymes, have offered new possibilities for addressing the challenges associated with metabolic diseases [26]. Compared with other materials, existing studies have shown that nanozymes have a strong potential in the management of metabolic diseases, mainly focusing on the following: (1) Nanozymes offer tunable properties such as size, shape, and surface chemistry that can be tailored to enhance their catalytic efficiency, targeting ability, and ability to eliminate ROS and alleviate oxidative stress, thereby promising potential for preventing the advancement of metabolic disorders and enhancing treatment efficacy, ultimately leading to better health outcomes [27, 28]. (2) Nanozymes can be designed to be biocompatible and biodegradable, reducing the risk of toxicity and ensuring safe use in biological systems for prolonged treatment, while the specific design and structural modification of nanozymes according to the characteristics of metabolic diseases will provide a new strategy for precise treatment [29]. (3) Nanozymes, through functionalization with specific ligands, provide targeted drug delivery systems that selectively bind to disease-related biomarkers and deliver therapeutic agents directly to the desired sites [30, 31]. (4) Nanozymes can be synthesized using cost-effective methods and materials, making them a more economically viable option for large-scale production and clinical translation. For example, Zhang et al. emphasize the multifunctional enzyme-like activities of nanozymes, such as antioxidant, oxygen-regulating, and NO-generating capabilities, which are crucial in alleviating diseases related to oxidative stress, and highlight the promising role of nanozymes in providing new treatment strategies for cardio- and cerebrovascular diseases [28]. Overall, although nanozymes have a relatively short development history, they have shown tremendous potential in the treatment and management of metabolic diseases.

In this review, the rational design strategies and cellular-level mechanisms of nanozymes in enabling therapeutic interventions are focused on. The application of nanozymes in treating various metabolic diseases, such as obesity, diabetes, cardiovascular disease, diabetic wound healing, and others, is extensively explored. Additionally, a comprehensive analysis of pharmacokinetics, safety considerations, challenges, and prospects regarding nanozyme application is presented. This review aims to provide valuable insights into nanozyme and their potential in advancing enzyme-mimicking approaches for metabolic disease therapy.

## Rational design strategies

### Design of enzyme-like activity

**Fig. 1:**
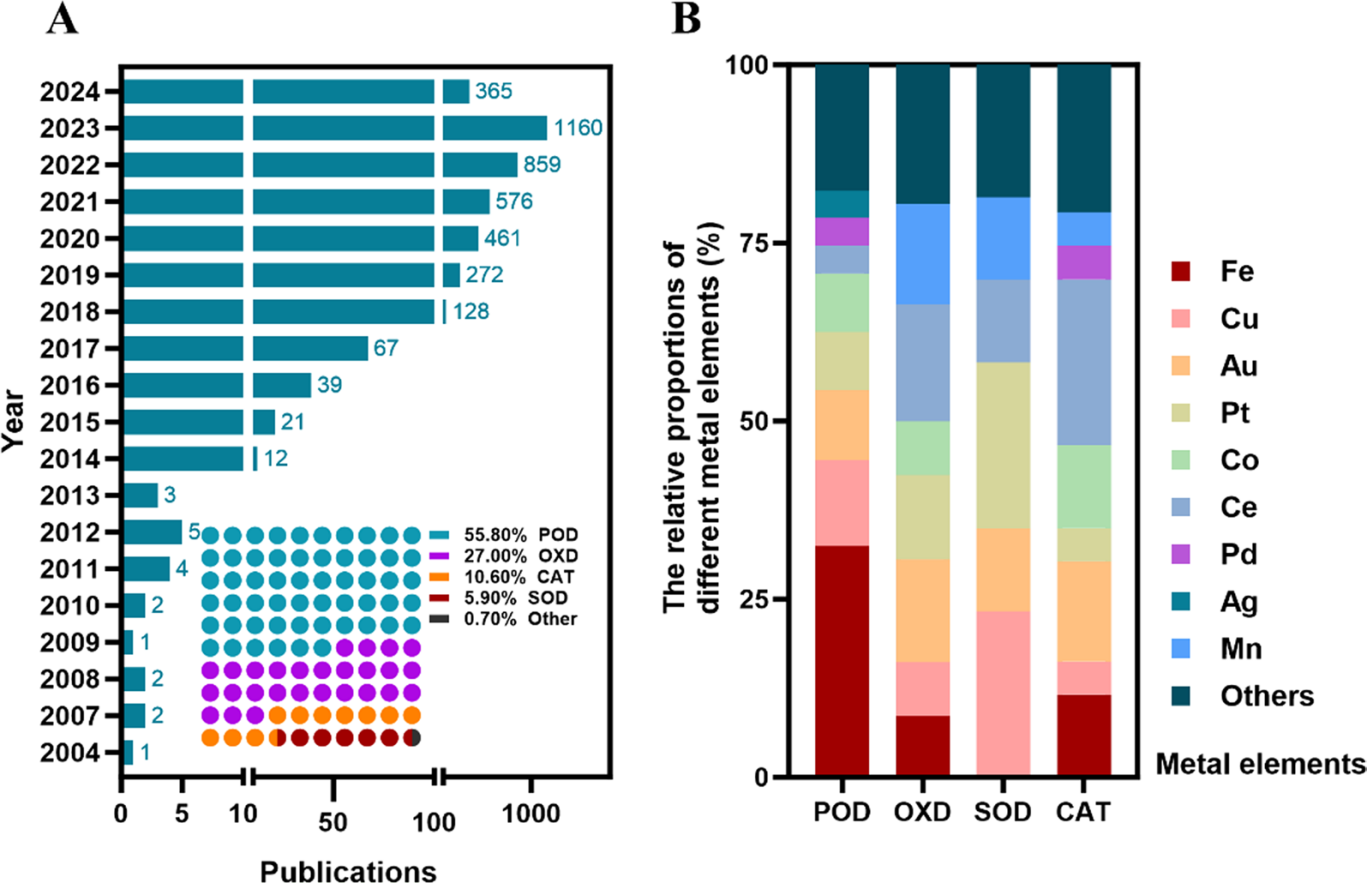
Research progress in the field of nanozyme. **A** Trends in the publication of nanozyme-related papers and the distribution of the types of nanozyme based on the Pubmed database until March 2, 2024. **B** Major metal elements in the active site of POD-; OXD-; SOD-; CAT-like nanozymes

Nanozymes possess a wide range of catalytic activities, making them highly valuable. Similar to natural enzymes, nanozymes can perform different types of catalysis: oxidoreductase, hydrolase, lyase, and isomerase-like catalysis. A series of studies have reported that dysregulation of these enzyme activities can lead to imbalances in cellular redox status and impact various metabolic processes, such as glucose and lipid metabolism, as well as carbohydrate metabolism. These disruptions have been implicated in metabolic diseases such as diabetes, obesity, and cardiovascular diseases [32–34]. For example, Otaki et al. firstly reported that plasma xanthine oxidoreductase activity with severity and clinical outcome in patients with chronic heart failure [35]. ATP citrate lyase (ACLY), an oxidoreductase that connects carbohydrate and lipid metabolism, converts citrate to acetyl-CoA, a crucial intermediate in fatty acid synthesis; thus, inhibiting ACLY activity can reduce fatty acid synthesis and potentially result in triglyceride accumulation along with a change in fatty acid composition in cancer cells. Abrogation of hepatic ACLY protects against fatty liver and ameliorates hyperglycemia in metabolic disorders mice [36, 37]. Among these, oxidoreductase-like nanozymes, such as POD-, superoxide dismutase (SOD)-, oxidase (OXD)-, and catalase (CAT)-like nanozymes, constitute over 96% of known nanozymes and have been extensively studied and developed [38]. As shown in Fig. 1, the existing studies suggest that the development of nanozymes has shown a spurt of growth and the metal elements often involved in the application of nanozymes in the management of metabolic diseases mainly include Fe, Cu, Au, Pt, Co, Ce, and etc. [39]. It is worth noting that in the past 10 years, the research on nanozymes has shown a blowout trend, and among the many nanozyme studies, POD-, OXD-, SOD-, and CAT-like activities have been the most widely studied. Besides, the surface functionalization of nanozymes refers to the process of modifying the surface of nanomaterials by incorporating specific functional groups or molecules, which enhance various properties of the nanozymes, such as catalytic activity, stability, selectivity, biocompatibility, and fulfill specific application needs. Recently, nanozymes containing atomically dispersed metal active centers have garnered significant attention for their potent catalytic and biological properties [40]. The presence of atomically dispersed active centers plays a crucial role in elucidating the relationship between structure and activity in nanozymes. By fine-tuning the composition [41], coordination environment [42], and heteroatomic doping [43] of these nanozymes, their catalytic efficiency, selectivity, and biological performance can be effectively controlled. In the following part, we will mainly focus on the design of nanozyme.

### Material selection

The material should possess high catalytic activity to efficiently mimic enzymatic reactions and allow for tuning of its catalytic properties, such as activity, selectivity, or stability, through modifications in its composition, structure, or surface properties. For example, the development of metal–organic frameworks (MOFs) with tailored structures and functional groups has shown promise in creating catalytic materials that can mimic enzymatic reactions. These MOFs can be designed to exhibit high catalytic activity, precise selectivity towards specific substrates, and enhanced stability under various reaction conditions, making them ideal candidates for catalyzing complex chemical transformations with efficiency and control [44, 45]. To ensure that the nanozymatic materials effectively mimic enzymatic reactions, Lu et al. designed a novel type of cobalt-doped carbon dots nanozymes. These were homogeneously doped with cobalt using a simple method, employing vitamin B12 and citric acid as precursors. The cobalt-doped carbon dots exhibited significant peroxidase-like activity, akin to natural metalloenzymes. This high catalytic activity endows the nano-enzyme with potential applications in glucose detection and tumor catalytic therapy [46].

Common materials used for nanozymes include metal nanoparticles (such as palladium, platinum, silver, or gold) [47–49], metal oxides (eg, manganese or cerium oxide) [50], carbon-based materials (carbon nanotubes or graphene) [51] and MOFs [52, 53]. Chen et al. have discussed the incorporation of MOFs into the active site of nanozymes, including OXD-, POD-, CAT-, and SOD-like nanozymes, as well as their representative nanozymes [39]. The specific material chosen will depend on the application requirements and the desired catalytic activity. For instance, researchers have developed a nanozyme by encapsulating L-arginine and ultrasmall gold nanoparticles within hyaluronic acid, and simultaneously loading Cu_1.6_O nanoparticles onto phosphorus-doped graphitic carbon nitride nanosheets. This nanozyme exhibits five distinct enzyme-like activities, namely POD-, CAT-, SOD-, nitric oxide synthase (NOS)-, and glucose oxidase (GOx)-like activities [54]. Understanding the catalytic mechanism of the target enzyme is essential for designing an enzyme-like activity in nanozymes. By studying the reaction pathway and identifying the key steps involved, researchers can design nanozymes that can mimic these steps and catalyze the same reaction.

### Surface functionalization

Surface functionalization of nanozyme involves modifying the surface of nanomaterials with specific functional groups or molecules to enhance their catalytic activity, stability, selectivity, or biocompatibility and to meet specific application requirements. For example, by attaching thiol groups onto the nanozyme surface, which can enhance its catalytic activity and stability [55]. The process of attaching thiol groups involves functionalizing the nanozyme surface with a linker molecule that has a reactive group capable of bonding with both the nanozyme and the thiol group [55]. This linker molecule acts as a bridge to covalently attach the thiol group onto the nanozyme surface. Once the thiol groups are successfully attached, they can interact with metal ions present in the environment or substrate. This interaction can lead to enhanced catalytic activity due to the formation of metal-thiol complexes that facilitate the enzymatic reactions [56]. To enhance the affinity of nanozymes to specific cellular receptors, ligands that are biocompatible and targeting, such as folate, transferrin, or RGD peptides, can be selected [57–59].

In addition, nanozymes can be coated with polymer layers to modify their surface properties. For example, poly (D, L-lactide-co-glycolide) (PLG) and poly (lactic-co-glycolic acid) (PLGA) are widely utilized biomaterials in the preparation of nanozymes due to their remarkable biodegradability, biocompatibility, and ease of chemical modification [31, 60, 61]. Polydopamine (PDA) is a widely recognized coating material that can undergo self-polymerization and spontaneously adhere to the surface of various materials, thus enabling the development of multifunctional nanomaterials [62]. Some researchers have discovered that PDA can serve as effective nanocarriers for DNA through conjugation facilitated by polyvalent metal coordination [63, 64]. In addition, nanozymes can be functionalized with biomolecules, such as enzymes, antibodies, or DNA, to impart specific catalytic or targeting properties [31, 65, 66]. This can be achieved through bioconjugation techniques, where the biomolecules are covalently attached to the nanomaterial surface. Therefore, nanozyme surface functionalization provides stability, prevents aggregation, improves dispersibility, and introduces specific functional groups for enhanced catalytic activity or biocompatibility.

### Size and shape control

The catalytic properties of nanozymes can be significantly influenced by their size and shape. When evaluating the in vivo performance of a nanozyme, important characteristics to consider include penetration into the target organ or tissue, concentration, particle size, and cellular absorption. Studies have indicated that smaller nano-particles (30–50 nm) may be cleared more rapidly from the tissue compared to larger ones (60–200 nm) [67, 68]. Hence, while the small size of nanoparticles facilitates improved tissue penetration, reducing their size often results in a shorter half-life in the bloodstream and rapid elimination from the body. The contradictory requirements regarding particle size have paved the way for the development of multi-stage size-switching systems. These include approaches such as removing the outer layer of nanoparticles, utilizing surface-carrying strategies, and employing nanoparticle-masking strategies, which aim to maintain broader initial blood circulation volumes [69, 70]. Generally speaking, nanozymes in the size range of 1 to 100 nm exhibit enhanced catalytic ability due to their increased specific surface area to volume ratio. This size parameter provides them with a vast surface area filled with active sites that can catalyze a large number of biochemical reactions with improved catalytic efficiency. For example, Xi et al. observed size-dependent POD-type properties in Pd-Ir nanoparticles ranging from 3.3 to 13.0 nm. Using an enzyme-linked immunosorbent assay as a model platform, they linked the enhanced catalytic properties of smaller nanoparticles to their diffusivities and reduced steric effects [71]. Additionally, these nanozymes are biocompatible and can be seamlessly incorporated into biological matrices. This property makes nanoparticles suitable for various biomedical applications, such as targeted drug delivery, biosensing devices, and therapeutic solutions [72]. Furthermore, the regulatory capabilities of these nanozymes enable easy functionalization by incorporating various molecular clusters, such as specific targeting ligand. This enhances their operational performance and ensures stability in different biological environments. This flexibility in functionalization allows for tailoring of the nanozymes to meet specific application requirements [73]. In addition, the small size of these entities allows them to penetrate cellular barriers and tissue structures with ease, ensuring unobstructed access to the desired target location. This ability is crucial for the effectiveness of targeted drug delivery systems and imaging technologies.

Overall, the design of enzyme-like activity in nanozymes involves selecting an appropriate nanomaterial, functionalizing its surface, understanding the catalytic mechanism, optimizing the nanozyme, and applying it in specific applications. This field holds great potential for developing novel catalytic systems with improved performance and broad applicability.

### Organ-specific targeting strategies

The design of organ-specific targeting for nanozyme involves the development of strategies to direct these nanomaterials toward specific organs in the body. This can be achieved through various approaches, including surface modification, functionalization, and encapsulation techniques [74, 75]. By modifying the surface of nanozymes with targeting ligands that specifically bind to receptors overexpressed on the target cells, they can be guided to interact with specific receptors or markers present in the cells of the target organ, which enhances the specificity of nanozyme delivery and reduce off-target effects [76, 77]. For example, nanozymes modified with silica nano-shells, polyethylene glycol-thiol shells, folic acid, and galactosylated ligands could target the liver and reduce side effects on other organs [30, 78–81]. In addition, engineer nanozymes to be internalized selectively by target cells through receptor-mediated endocytosis. This can enhance the uptake of nanozymes by the desired cells while reducing non-specific interactions with normal cells [82]. Incorporate magnetic nanoparticles into nanozymes and use an external magnetic field to guide their accumulation at the target site. This approach can improve the localization of nanozymes and reduce systemic exposure [83]. Furthermore, the optimization of nanozymes’ size, shape, and surface charge can also improve their accumulation and retention specifically in the targeted organ [84]. Overall, organ-specific targeting of nanozymes holds great potential for improving the safety and efficacy of therapeutic interventions. In the following parts, we will focus on exploring various strategies for organ targeting, coupled with the fact that the liver, adipose, and pancreas act a vital role in the metabolism of the body, with the aim of providing new ideas for designing diverse nanozymes that can effectively manage metabolic diseases in the future (Table 1).

**Table 1:** The specific targeted strategies for different organs

Organ	Targeted strategies	References
Liver	Silica nano-shells, polyethylene glycol-thiol shell, folic acid, and galactosylated ligands	[30, 78–81]
Adipose	The peptide CSWKYWFGEC and the peptide KGGRAKD	[66, 85]
Pancreas	RNA aptamers and exosomes	[86–88]

### Liver

The liver is a vital organ involved in various metabolic processes, drug metabolism, and detoxification [89, 90]. Therefore, liver-specific nanozymes hold great potential for applications in liver-targeted drug delivery, liver disease diagnosis, and therapeutic interventions. Surface modifications or functionalization of nanozymes can be used to specifically target and bind to receptors or markers present on liver cells, thereby enabling targeted delivery and enhancing therapeutic efficacy. For example, these liver-specific biodegradable silica nano-shells, encapsulating platinum nanoparticles (Pt-SiO_2_), function as ROS scavengers and also serve as functional hollow nanocarriers. To achieve prolonged and efficient removal of ROS in the liver tissue of T2D models, a strategy involves loading 2,4-dinitrophenol-methyl ether inside Pt-SiO_2_ nanostructures and subsequently applying a lipid bilayer coating (D@Pt-SiO_2_@L) [78]. Similarly, scientists have devised a carefully engineered micelle system comprising an α-tocopheryl hydrophobic core and a detachable polyethylene glycol-thiol shell. This design aims to target the liver for efficient deposition of berberine, ensuring its effectiveness within the organ [30]. Folic acid (FA) serves as a tumor-targeting ligand, selectively binding to folate receptors overexpressed on the surface of cancer cell. Liu et al. reported that ZnMnFe_2_O_4_-PEG-FA nanoplatform possessing photothermal and POD-like activity was developed for tumor therapy via synergistic tumor cell diagnosis and ablation [79].

In addition, the asialoglycoprotein receptors (ASGPR) exhibit high levels of expression in hepatic parenchymal cells while demonstrating minimal expression in non-hepatic cells. Studies have demonstrated that galactosylated ligands exhibit a high affinity for ASGPR and can effectively recognize and bind to these receptors [80, 81]. As a result, galactosylated nanoparticles present an appealing strategy for hepatic drug delivery, as they can specifically target the ASGPR receptors expressed on hepatocytes. Teng and colleagues have successfully developed hepatic-specific nanocarriers by conjugating oxidized starch-lysozyme with galactose. This innovative method allows for the targeted delivery of resveratrol to the liver, presenting a promising and safe strategy for treating liver-related conditions, such as nonalcoholic fatty liver disease [91]. Similarly, liver-targeted Ce-based hollow mesoporous nanocarriers, covalently linked with galactose and loaded with resveratrol, were developed for the precise relief of nonalcoholic steatohepatitis [92]. The development of liver-specific nanozyme offers encouraging prospects for enhancing the management of liver-related diseases and reducing adverse effects.

### Adipose

Adipose tissue is a crucial energy storage and metabolic organ, and targeting it with nanozyme can have implications for obesity, metabolic disorders, and related diseases. Different strategies can be employed to achieve specific targeting of adipose tissue for effective treatment. One strategy is to modify the surface of the nanozymes with ligands or antibodies that have an affinity for receptors or markers expressed on adipose cells. For example, the peptide CSWKYWFGEC (AHP) has been documented to exhibit selective binding to the non-glycanated decorin on adipose stromal cells within white adipose tissue. Consequently, it has been extensively employed as a means to specifically deliver drugs or nanoparticles to adipocytes [66]. Similarly, the peptide KGGRAKD, referred to as the adipocyte-targeting sequence (ATS), has been identified as a ligand capable of specifically binding to prohibitin, a receptor that is found to be overexpressed on the plasma membrane of adipocytes [85]. To enable receptor-mediated endocytosis by adipocytes, the ATS peptide is chemically linked to PLG nanoparticles, thus acting as an active targeting ligand [93]. Han et al. showcased the synthesis of gas-generating nanoparticles (GNP) by incorporating CaCO_3_ into PLG nanoparticles through a double emulsion technique. To facilitate adipocyte-specific endocytosis, they chemically attached ATS peptide onto the surface of PLG nanoparticles, leading to the formation of ATS-GNP nanoparticles [31]. Xue et al. developed two peptide-functionalized nanoparticles (NPs) platforms by self-assembling a biodegradable triblock polymer consisting of poly (lactic-co-glycolic acid)-b-poly (ethylene glycol) (PLGA-b-PEG) with end-to-end linkages and an endothelial-targeted peptide, which enhances the targeted delivery of nanoparticles to angiogenic vessels within adipose tissues [94]. Taken together, by incorporating these strategies, organ-specific targeting of adipose nanozyme can be achieved, enabling potential utilizations in targeted drug delivery, imaging, and therapeutic interventions for adipose-related disorders.

### Pancreas

The pancreas has a vital role in metabolic disorders, especially in conditions like diabetes. Enhancing our understanding and treatment of diabetes could significantly benefit from the advancement of techniques capable of detecting and selectively targeting β cells within the body. Nevertheless, the current absence of specific probes hinders our ability to accurately measure the mass of human β cells and deliver effective treatments in clinical settings. Van et al. have documented the discovery of two RNA aptamers, which selectively and specifically recognize mouse and human β cells by targeting transmembrane p24 trafficking protein 6 and clusterin [86]. Multiple investigations have showcased the effectiveness of utilizing exosomes, which have emerged as highly promising nano-carriers for delivering therapeutic agents like small interfering RNA, microRNAs, proteins, or chemical medicines to targeted cells [87, 88]. A technique was developed to synthesize exosomes loaded with BAY55-983 and coupled with superparamagnetic iron oxide nanoparticles. This innovative approach resulted in engineered exosomes with enhanced targeting ability towards pancreas islets and prolonged plasma half-life time compared to free BAY55-9837 [95]. Notably, a novel nanozyme (PtFe@Fe_3_O_4_) with dual enzyme-like activities for highly efficient tumor catalytic therapy. PtFe@Fe_3_O_4_ shows the intrinsic photothermal effect as well as photo-enhanced POD-like and CAT-like activities, thereby effectively targeting pancreas killing tumor cells and overcoming the tumor hypoxia [96]. Taken together, pancreatic targeting of nanozymes can be achieved by different designs.

### Real-time and in-situ detection design for metabolic diseases

Significant efforts have been dedicated to the progress of miniature analytical systems for monitoring glucose levels in blood or urine samples in vitro, which hold great importance in the management and control of diabetes [97, 98]. Microfluidics has gained significant traction as a highly effective tool, with wide-ranging applications in the domain of biomedical research owing to its numerous advantages, such as enhanced portability, reduced sample requirements, and accelerated detection time [99]. The use of microfluidic devices based on nanozymes has gained substantial interest in the field of biomedical research focusing on metabolic disease due to their potential to offer a simple, rapid, and easily transportable approach [100]. For example, simultaneous immobilization of NiPd hollow nanoparticles and GOx onto zeolitic imidazolate framework 8 (GOx@ZIF-8(NiPd)) results in the creation of nanoflower-shaped structures that exhibit POD-like activity and can function as glucose sensors [101]. Piao et al. developed a novel glucose monitoring system utilizing water-in-air droplets within a microfluidic device incorporated with GOx. This innovative droplet-based glucose sensing chip enables the real-time, in-situ, and high-throughput monitoring of glucose levels [102]. Similarly, a rudimentary hydrogel nanozyme containing a single Fe–N site, exhibiting POD-like activity, was integrated with GOx into a foldable paper microfluidic device. This portable and user-friendly tool enables on-site glucose detection, paving the way for potential diabetic diagnosis in instrumentation-free point-of-care testing environments [103].

Metabolic diseases are a group of disorders that involve abnormalities in the body’s energy conversion and metabolic pathways, including but not limited to diabetes, obesity, hyperlipidemia, and NAFLD. These diseases are usually caused by a variety of factors including genetics, diet, lifestyle and environmental factors. Therefore, in addition to blood glucose levels, there are many other biomarkers and physiologic parameters that can indicate the presence and progression of metabolic diseases, such as, insulin levels, lipid profile, liver function markers, inflammatory markers, and hormone levels. By monitoring these multiple parameters, we can more comprehensively assess an individual's metabolic health and provide more accurate information for early diagnosis and personalized treatment. In summary, combining suitable nanozymes and microfluidics enables the in situ and real-time detection of glucose levels in blood and urine, providing vital diagnostic measures for diabetes and other metabolic diseases.

## The therapeutic mechanism of nanozymes in metabolic disease

### Modulation of glucose uptake

**Fig. 2:**
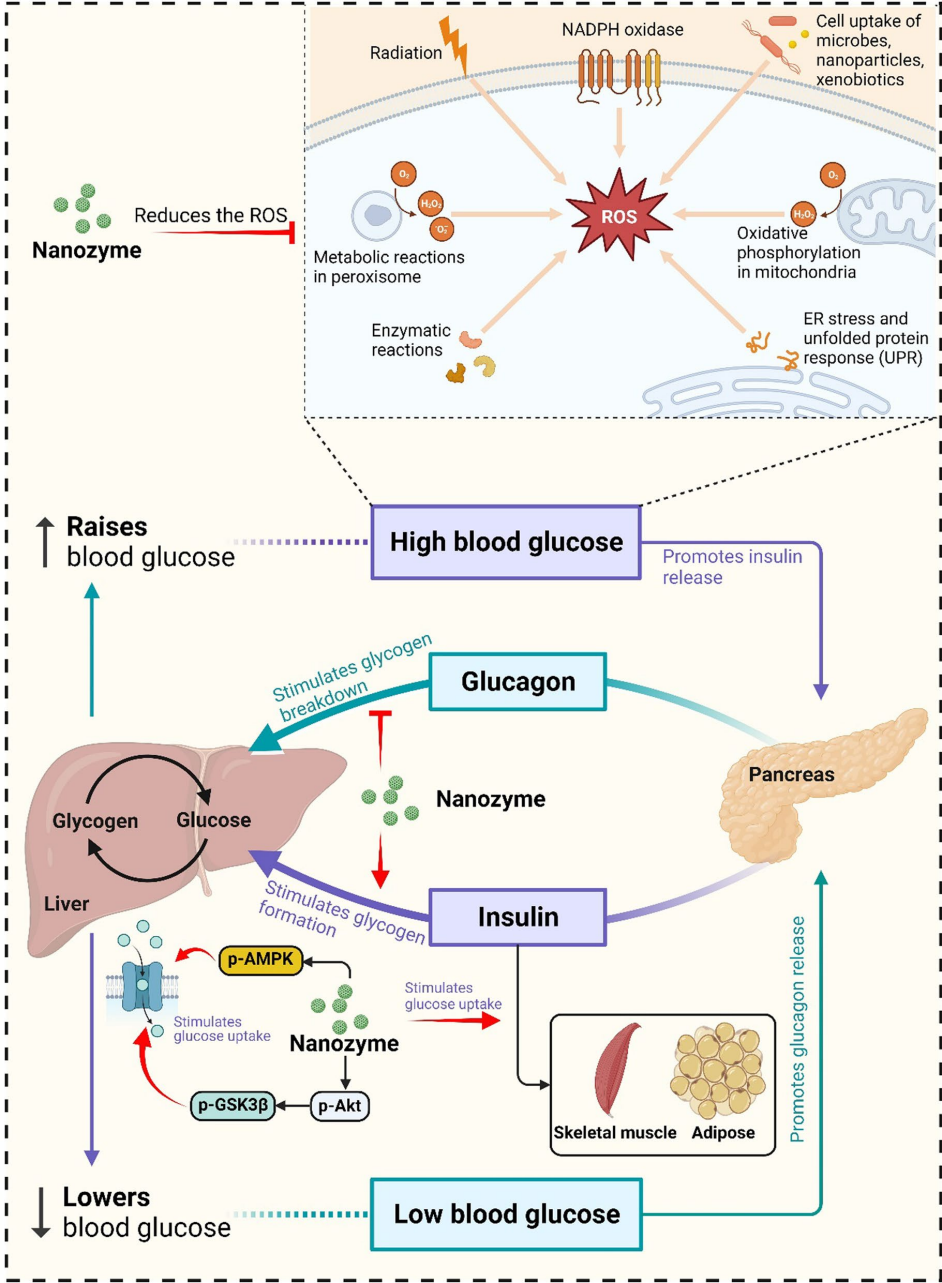
The therapeutic mechanism of nanozymes in metabolic disease. Nanozyme modulates metabolic homeostasis by increasing glucose uptake and insulin sensitivity, stimulating glycogen synthesis, reducing inflammation and insulin resistance, and oxidative stress

Glucose uptake refers to the process by which glucose is taken up by cells in the body for energy production. In individuals with diabetes, there are issues with glucose uptake, leading to elevated blood glucose levels (Fig. 2). Iron oxide nanoparticles (Fe_3_O_4_ NPs), a widely recognized type of nanozyme, have been shown to produce ROS at levels close to physiological concentrations within lysosomes. This local increase in ROS leads to the activation of adenosine 5’-monophosphate-activated protein kinase (AMPK), thereby enhancing the glucose uptake capacity in both normal cells (L6 myoblast cells, HepG2 hepatocytes, and 3T3-L1 preadipocytes) and insulin resistant cells (L6 myoblast cells, HepG2 hepatocytes) [104]. Based on our previous investigation, we observed that the signal-atom Ce-N_4_-C-(OH)_2_ nanozyme was predominantly localized within lysosomes, resulting in the generation of •OH and the subsequent enhancement of glucose uptake in HepG2 cells [105]. Furthermore, in a separate study by Chang et al., it was demonstrated that Cu single-atom nanozyme loaded with licogliflozin exhibited remarkable multienzyme activities, leading to the generation of a ROS storm. This, in turn, resulted in the inhibition of glucose uptake by blocking the “valve” of the sodium-dependent glucose transporter, effectively disrupting the energy source required for ATP generation [106].

### Facilitation of glycogen synthesis

Glycogen is a complex carbohydrate that serves as the primary storage form of glucose in animals and humans [107]. When blood glucose levels are elevated, hepatocytes (liver cells) uptake the surplus glucose and convert it into glycogen through a metabolic process known as glycogenesis [108]. Recently, studies have shown that certain nanozymes play a significant role in facilitating glycogen synthesis by leveraging the collective enzymatic activity of multiple enzymes. For example, the signal-atom Ce-N_4_-C-(OH)_2_ nanozyme demonstrated activities similar to SOD-, OXD-, CAT-, and POD counteracting substrate limitations and generating •OH. This led to local activation of protein kinase B phosphorylation (p-Akt), which further stimulated glycogen synthase kinase 3β phosphorylation and glycogen synthase expression. Consequently, this promoted glycogen synthesis, thereby improving systemic glucose homeostasis [105].

### Increased insulin sensitivity and reduced insulin resistance

Insulin sensitivity is defined as the capacity of cells to effectively respond to insulin and uptake glucose from the bloodstream [109]. Conversely, insulin resistance refers to a condition where cells exhibit reduced responsiveness to insulin, resulting in elevated levels of glucose in the bloodstream. Both insulin sensitivity and insulin resistance are significant factors in the development and management of diabetes [110]. Nanozymes have shown promise in improving insulin sensitivity and reducing insulin resistance. They can mimic the action of natural enzymes involved in glucose metabolism, including GOx and CAT. These nanozymes can enhance the breakdown of glucose and regulate its levels in the bloodstream. Several studies have demonstrated the potential of nanozymes in reducing insulin resistance and improving insulin sensitivity [104, 105]. By reducing oxidative stress and inflammation, these nanozymes can enhance insulin sensitivity and improve glucose metabolism [78]. Furthermore, nanozymes can also be used to deliver insulin or other antidiabetic drugs to the target sites more effectively. The compound D@Pt-SiO_2_@L has been shown to effectively reduce oxidative stress, insulin resistance, and glucose uptake impairment in cell studies. In diabetic mouse models induced by a high-fat diet (HFD) and streptozotocin, D@Pt-SiO_2_@L significantly improves liver fat accumulation and antioxidant capacity. Furthermore, intravenous administration of D@Pt-SiO_2_@L demonstrates therapeutic benefits for hyperlipidemia, insulin resistance, hyperglycemia, and diabetic nephropathy. These findings suggest that D@Pt-SiO_2_@L could be a promising treatment for type 2 diabetes by reversing liver insulin resistance through continuous scavenging of ROS [78]. These nanozymes can be engineered to deliver insulin in a controlled manner, enhancing its bioavailability and mitigating the potential for adverse effects [27]. Overall, nanozymes hold great potential in improving insulin sensitivity and reducing insulin resistance in diabetes.

### Anti-inflammation and anti-oxidation

Studies have demonstrated that oxidative stress and chronic low-grade inflammation are key mechanisms that link metabolic comorbidities [12, 111]. ROS are active small molecules like hydroxyl radical (•OH), superoxide anion radical (•O_2_^−^), hydrogen peroxide (H_2_O_2_), and hypochlorous acid (HClO), which contribute to and worsen inflammation. Over the past few decades, the utilization of nanozymes to disrupt the feedback loop between oxidative stress and inflammatory pathways has been recognized as a potentially promising strategy. For example, Parra-Robert and coworkers found that mesoporous silica-coated CeO_2_ (CeO_2_@mSiO_2_) nanozyme performs a pivotal role in down-regulating the phosphoinositide 3-kinase (PI3K)/ mammalian target of Rapamycin (mTOR)/AKT pathway and reducing hepatic M1 proinflammatory cytokine tumor necrosis factor-α (TNF-α), which are critical in the regulation of systemic metabolism, lipid homeostasis, and inflammation [112]. It is widely acknowledged that anti-inflammatory effects are typically accompanied by antioxidative effects. Studies have shown that the MnCoO@PLE/HA hydrogel possesses the capability to modulate macrophage polarization from M1 to M2 by scavenging ROS and generating oxygen. This contributes to the resolution of excessive inflammation [113]. Similarly, treatment with a microenvironmentally adaptive nanohybrid double network hydrogel (PFOB@PLGA@Pt/GelMA/ODex) has the potential to effectively reduce inflammation through its ability to exhibit elevated levels of interleukin 10 and reduced levels of CD86, primarily through its SOD- and CAT-like activities [114]. Simvastatin@ porous manganese-substituted Prussian blue (PMPB) NC could effectively scavenge excessive ROS under HFD conditions. Based on this finding, it is expected that Simvastatin@PMPB NC can also contribute to the mitigation or reduction of inflammation. Additionally, the study revealed that Simvastatin@PMPB NC significantly suppressed inflammation-associated cytokines secretion, such as TNF-α, interleukin 6, and monocyte chemotactic protein-1, further emphasizing its anti-inflammatory properties [115]. CeO_2_@mSiO_2_ nanoparticles have been shown to enhance the expression of genes associated with a downregulation of the hepatic PI3K/mTOR/AKT pathway and a decrease in the proinflammatory cytokine TNF-α in liver and adipose tissue. Further investigation is required to elucidate the precise molecular mechanisms that establish the connection between the accumulation of CeO_2_ nanomaterials in the liver and their protective effects observed in serum and adipose tissue [112]. Generally speaking, nanozyme exerts anti-inflammatory and antioxidant effects through its enzyme-like activity, providing an important foundation for its clinical applications. The physiological functional mechanism of nanozymes in regulating inflammation and oxidative stress needs to be extended in vivo using conditional or whole-body knockout mice.

## The application of nanozyme in metabolic disease management

Recently, as the research on nanozyme continues to advance, they have shown significant competitiveness in the management of metabolic disorders. This section will predominantly concentrate on exploring the wide-ranging applications of nanozymes in the treatment and control of metabolic disorders, encompassing obesity, diabetes, cardiovascular disease, and the healing of diabetic wounds, encompassing multiple perspectives (as presented in Table 2).

**Table 2:** The application of nanozyme in metabolic disease management

Type of diseases	Nanozymes	Method of administration	In vitro or in vivo models	Mechanism	Effects	Reference
Obesity	CeO_2_@mSiO_2_	Intravenous injection	• HepG2 hepatocytes • Zucker rats	• Reduce ROS levels • Reduce the M1 proinflammatory cytokine TNF-α • Down-regulation of the hepatic PI3K/mTOR/AKT pathway	• Antioxidant activity and cellular protection • Reduce circulating levels of TNF-α, triglycerides, palmitic acid, and LDL-cholesterol	[112]
	Apt-Au_25_ NCs	NA	• 3T3-L1 preadipocytes	• SOD- and CAT-like activity • Scavenge ROS in white adipocytes	• Antioxidant activity	[116]
Diabetes	Fe_3_O_4_ NPs	Intraperitoneal injection	• L6 myoblast cells, HepG2 hepatocytes and 3T3-L1 preadipocytes • *Drosophila melanogaster* • *Ob*/*ob* mice	• POD- and CAT-like activity • Generate nearly physiological levels of ROS within the compartment of lysosomes • Induce AMPK activation	• Enhance glucose uptake • Reduce the levels of circulatory sugar and hyperinsulinemia • Lower blood sugar levels, and improve glucose and insulin tolerance	[104]
	SACe-N_4_-C-(OH)_2_	Intraperitoneal injection	• HepG2 hepatocytes • HFD-induced diabetic mice	• SOD-, OXD, POD- and CAT-like activity • Improve •OH production in HepG2 cells • Activate the phosphorylation of AKT and promote the expression of pGSK3β and GS	• Enhance glucose uptake • Lower blood glucose levels • Alleviate glucose tolerance and insulin resistance	[105]
Cardiovascular disease	Simvastatin@PMPB	Intravenous injection	• RAW264.7 cells • ApoE^−/−^ mice	• SOD- and CAT-like activity • Scavenge ROS and mitigate inflammation	• Decrease oxidative stress, macrophage infiltration, plaque density, LDL internalization, fibrous cap thickness, and foam cell birth	[115]
	PtCe NRs	Intravenous injection	• RAW264.7 cells • ApoE^−/−^ mice	• SOD- and CAT-like activity • Scavenge ROS	• Weaken the plaques via synergistic foam cell inhibition and antiplatelet aggregation • Reduce the production of oxidized low-density lipoproteins in plaques • Inhibit the formation of foam cells	[117]
Diabetic wound healing	MnCoO@PLE	Transdermal patch	• Skin cells • STZ-induced diabetic rats	• Scavenge ROS in diabetic wounds • Promote oxygen production • Induce the macrophages polarization from M1 to M2 type	• Alleviate the excessive inflammatory • Induce efficient proliferation, re-epithelialization, collagen deposition, and neovascularization	[113]
	Hyperbranched poly-L-lysine-modified MnO_2_	Transdermal patch	• Methicillin-resistant *Staphylococcus aureus* • Mouse L929 fibroblasts • HFD fed-male GotoKakizaki (GK) rats	• Scavenge ROS and generate O_2_ • Reduce the level of neutrophil infiltration • Enhance M2-type macrophage polarization	• Kill broad-spectrum bacteria, and protect cells against oxidative stress • Stimulate neovascularization and deposition of collagen with a thicker skin and epithelium structure	[118]
	MoS_2_@Au@BSA NSs	Transdermal patch	• STZ-induced female diabetic rats	• SOD-, CAT-, GOx-, and POD-like activity • Scavenge ROS • Reduce oxidative stress, alleviate hypoxia, and facilitate glucose oxidation	• Accelerate wound closure by antibacterial ability, antioxidant activity, O_2_ provision, and human umbilical vein endothelial cell proliferation and migration	[119]
	ACPCAH	Transdermal patch	• *Staphylococcusaureus*, multidrug-resistant *Staphylococcusaureus*, *Escherichia coli*,* Pseudomonas aeruginosa* • RAW264.7 cells • Human skin fibroblast cells • STZ-induced diabetic rats	• SOD-, CAT-, GOx-, POD-, and NOS-like activities • Anti-bacterial and anti-inflammation	• Reduce inflammation • Relieve hypoxia • Lower blood glucose • Promote angiogenesis • Eliminate pathogenic bacteria	[54]
	PFOB@PLGA@Pt	Transdermal patch	• *Staphylococcusaureus* • Human umbilical vein endothelial cells • Mouse L929 fibroblasts • RAW264.7 cells	• NADH oxidase-, POD-, OXD-, CAT-, and SOD-like activities • Scavenge ROS	• Ameliorate the hypoxia • Synergistic antibacterial effects • Reshape the redox microenvironment	[114]
	Fe_3_O_4_-GOx	Transdermal patch	• Methicillin-resistant *Staphylococcus aureus* and *Escherichia coli* (*E. coli*) • Human umbilical vein endothelial cells • *db*/*db* mice	• GOx, CAT, and POD-like activities • Inhibit excessive oxidative stress	• Reduce the inflammatory phase and initiate normal wound-healing processes • Accelerate the proliferation and remodeling phases of wound healing	[120]

*ACPCAH* Hyaluronic acid encapsulated L-arginine and ultrasmall gold nanoparticles and Cu_1.6_O nanoparticles coloaded phosphorus doped graphitic carbon nitride nanosheets, *AKT* Protein kinase B, *AMPK* Adenosine 5'-monophosphate (AMP)-activated protein kinase, *CAT* Catalase, *GOx* Glucose oxidase, *GS* Glycogen synthase, *HFD* High-fat diet, *LDL* Low-density lipoprotein, *NA* Not applicable, *NADH* Nicotinamide adenine dinucleotide, *NOS* nitric oxide synthase, *OXD* Oxidase, *POD* Peroxidase, *pGSK3β* Phospho-glycogen synthase kinase3β, *ROS* Reactive oxygen species, *SOD* Superoxide dismutase, *STZ* Streptozotocin, *T2D* Type 2 diabetes, *TNF-α* Tumor Necrosis Factor-α

### Obesity

**Fig. 3:**
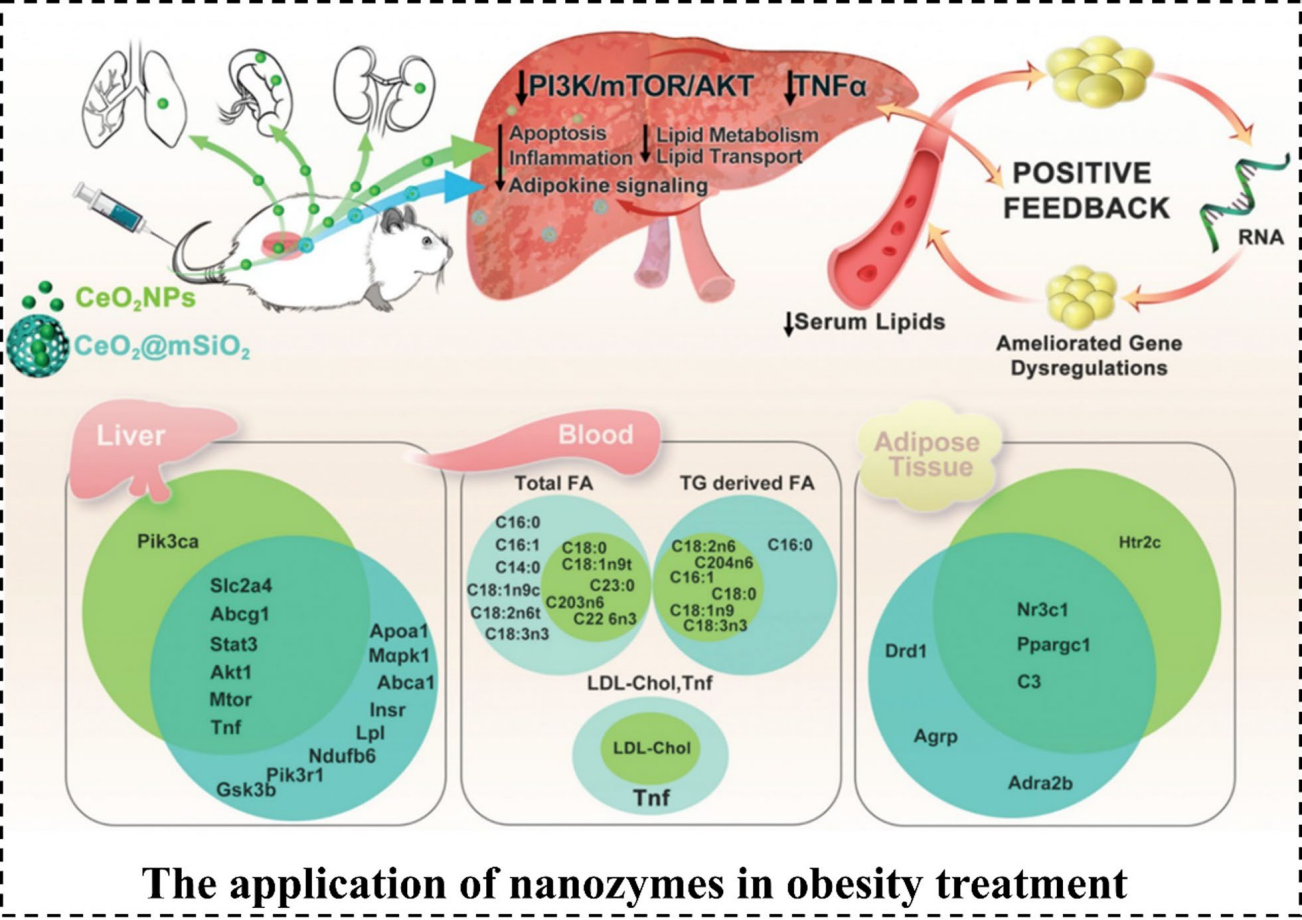
The application of nanozymes in obesity treatment. By addressing the low-grade chronic inflammation and oxidative stress associated with obesity, CeO_2_ nanoparticles (CeO_2_NPs) and mesoporous silica-coated CeO_2_ (CeO_2_@mSiO_2_) induce long-term improvements in the metabolic profile. Reproduced with permission [112]. Copyright 2021, The Royal Society of Chemistry

Obesity is a prevalent metabolic disorder that is characterized by the abnormal and excessive accumulation of body fat, leading to detrimental effects on an individual’s overall health [121]. Previous studies have indicated that excessive levels of ROS contribute to oxidative stress, which not only disrupts the functionality of white adipocytes but also directly hampers adipocyte respiration [122, 123]. Consequently, this process facilitates the accumulation of triglycerides, which further leads to obesity. Recently, numerous nanozymes exhibiting remarkable ROS-scavenging abilities have been reported as potent antioxidants, demonstrating high efficacy in the treatment of obesity [124–126]. Notably, Ding et al. constructed aptamer-modified atomically precise gold Au_25_ nanoclusters (Apt-Au_25_ NCs) that exhibit high concentration-dependent CAT- and SOD-like activity. This targeted nanozyme, Apt-Au_25_ NCs, has shown high efficiency in eliminating ROS in white adipocytes, which demonstrates the promising potential for the management of obesity and related diseases [116]. Following that, the team developed a targeted delivery vehicle for the browning agent docosahexaenoic acid (DHA@Apt-NG) which notably enhanced the mRNA expression levels of *Prdm16*, *Pparg*, *Pgc-1α*, and *Ucp1*. This study presents a viable approach for achieving effective browning of white adipocytes using targeted delivery nano-systems, thereby providing novel insights for the treatment of obesity [127]. In addition, a novel nanocomposite utilizing mesoporous silica-coated CeO_2_ nanozymes was developed. When administered to obese Zucker rats, it effectively reduced circulating fatty acid levels and significantly improved the metabolic phenotype after five weeks. This enhancement was associated with the inhibitory regulation of the hepatic PI3K/mTOR/AKT pathway and a decrease in the expression of the proinflammatory cytokine TNF-α, along with improvements in hyperlipidemia and hepatic and adipose metabolic dysregulations as shown by lipidomic and gene expression analyses (Fig. 3) [112]. Taken together, the nanozymes mentioned above provide a new strategy for treating metabolic comorbidities associated with obesity.

### Diabetes

**Fig. 4:**
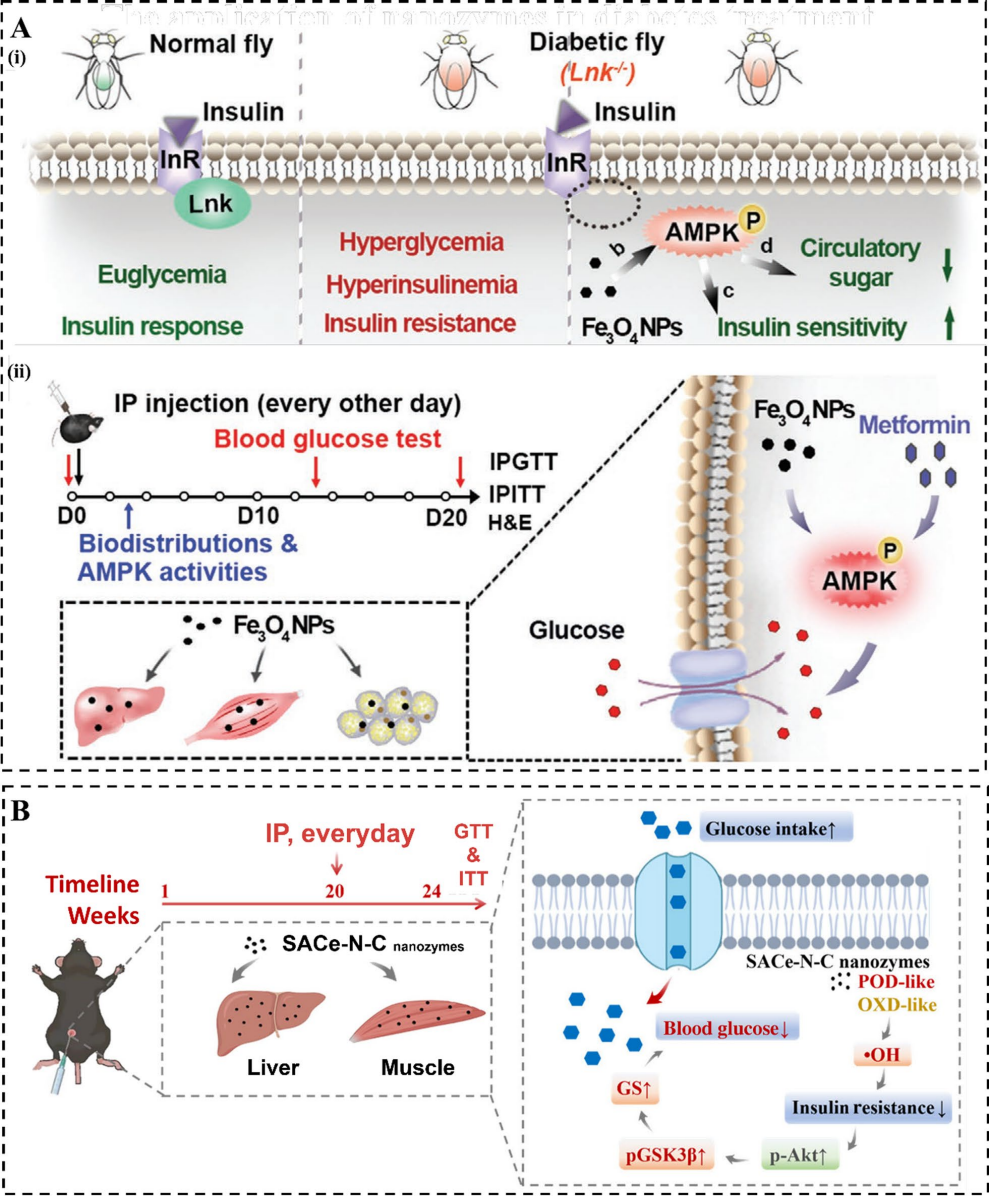
The application of nanozymes in diabetes treatment. **A** The effects of Fe_3_O_4_ NPs nanozyme in (i) *Drosophila* and (ii) *ob*/*ob* mouse models of T2D. Reproduced with permission [104]. Copyright 2020, Wiley–VCH GmbH. **B** The effects of single atom Ce-N_4_-C-(OH)_2_ nanozyme in diet-induced diabetes mouse models of T2D. Reproduced with permission [105]. Copyright 2023, the American Association for the Advancement of Science and China Association for Science and Technology

Diabetes is a chronic condition characterized by elevated levels of blood glucose, which can lead to detrimental effects on the heart, kidneys, eyes, blood vessels, and nerves as time progresses [128]. It occurs either due to insufficient insulin production by the pancreas or ineffective utilization of the insulin produced by the body. Metformin, sulfonylureas, and different types of insulin are the main drugs for clinical hypoglycemic treatment [129]. While these medications can effectively regulate blood glucose levels, they may also have potential adverse effects including pancreatic degeneration, gastrointestinal discomfort, hypoglycemia, and liver damage. The emergence of nanozymes has significantly influenced the field of diabetes diagnosis and treatment [130]. For example, Fe_3_O_4_ NPs have been found to stimulate the activation of AMPK and improve glucose uptake. Dietary administration of Fe_3_O_4_ NPs has positive effects in treating high blood glucose and excess insulin levels in genetic manipulation or a high-glucose diet-induced diabetes *Drosophila* model. Additionally, the intraperitoneal injection of Fe_3_O_4_ NPs led to an augmentation of AMPK activity of diabetic *ob*/*ob* mice, consequently leading to the improvement in insulin sensitivity, enhancement of glucose tolerance, and reduction of blood glucose levels (Fig. 4A) [104]. Subsequently, our team discovered for the first time that the single-atom Ce-N_4_-C-(OH)_2_ nanozyme exhibits POD-, CAT-, OXD-, and SOD-like activities within the liver and muscle tissues involved in glucose metabolism. This allows the nanozyme to overcome substrate limitations and independently generate •OH, resulting in a significant therapeutic effect in alleviating hyperglycemia, characterized by enhancing glucose uptake, promoting glycogen synthesis, alleviating insulin resistance, improving glucose tolerance, and enhancing overall systemic glucose homeostasis (Fig. 4B) [105]. Overall, these studies unveil the significant role of nanozyme in modulating glucose homeostasis and indicate their potential efficacy in diabetes care.

### Cardiovascular disease

**Fig. 5:**
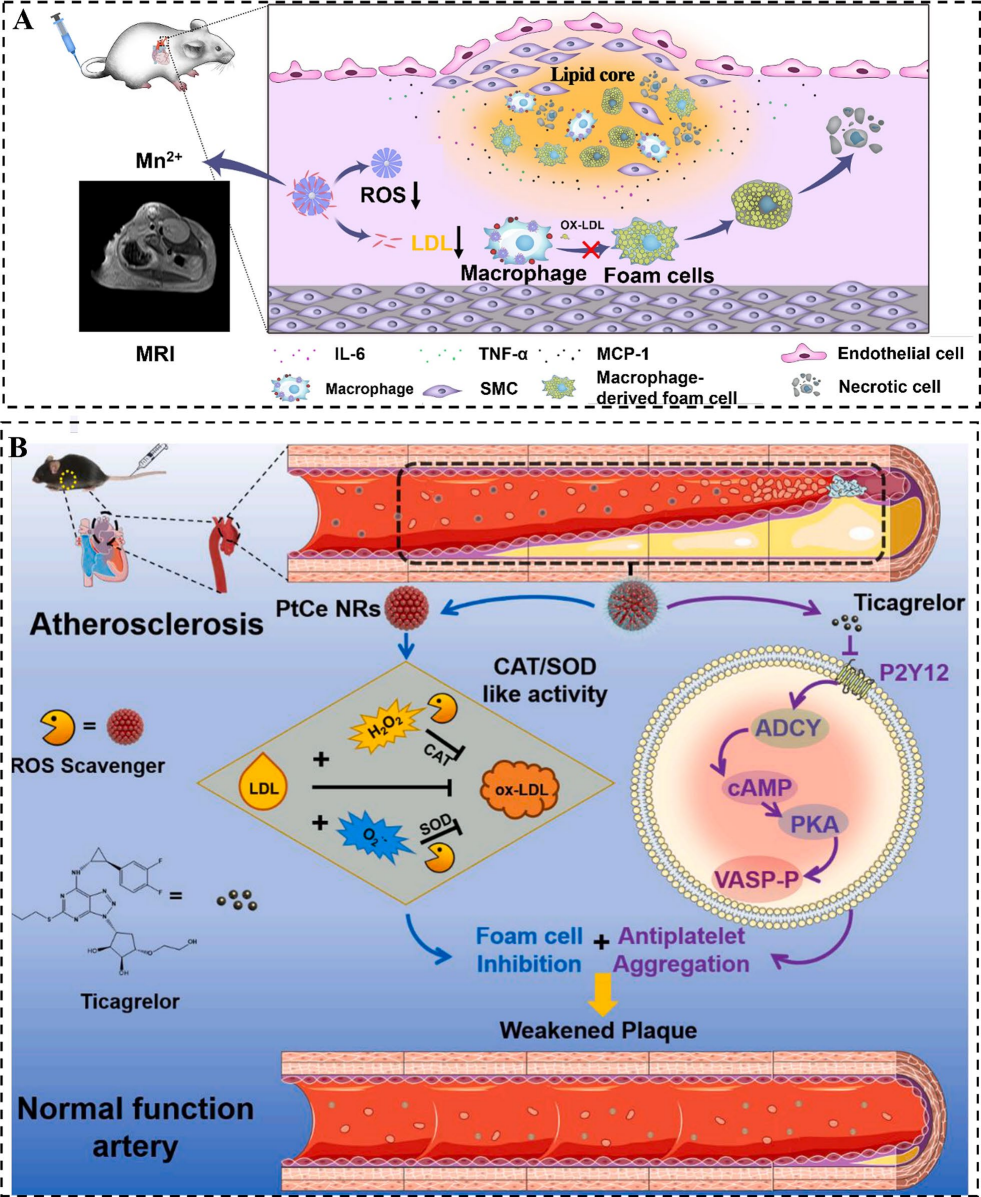
The application of nanozymes in cardiovascular disease treatment. **A** The underlying principle using Simvastatin@PMPB to treat atherosclerosis. Reproduced with permission [115]. Copyright 2020, Springer. **B** Platinum-cerium bimetallic nano-raspberry for atherosclerosis treatment via synergistic foam cell inhibition and P2Y12 targeted antiplatelet aggregation. Reproduced with permission [117]. Copyright 2020, Elsevier

Metabolic disorders such as glucose metabolism disorder, lipid metabolism disorder, and amino acid metabolism disorder are directly related to cardiovascular diseases including arrhythmia, heart failure, atherosclerosis, and ischemic heart disease [15]. Atherosclerosis, a prevalent cardiovascular condition, presents a substantial risk to personal health and overall well-being [131]. It is widely recognized that oxidative stress and inflammation act pivotal roles in the progression of atherosclerotic plaques, contributing to all stages of the disease [132]. The field of nanomedicine and nanotechnology presents significant promise in addressing the existing clinical needs of developing drugs for anti-oxidative and anti-inflammatory therapies aimed at combating atherosclerosis. For example, the use of a biomimetic theranostic agent loaded with simvastatin, utilizing PMPB analogues as carriers, exhibits remarkable efficacy in scavenging ROS and effectively reducing inflammation, as demonstrated in both in vivo and in vitro studies. The biomimetic simvastatin@PMPB theranostic agent demonstrates significant efficacy in promoting the stabilization of atherosclerotic plaques, resulting in the mitigation of atherosclerosis progression. It also enables the localization and magnification of atherosclerosis, facilitating the monitoring of the evolutionary changes in H_2_O_2_-associated atherosclerosis after treatment (Fig. 5A). Moreover, simvastatin@PMPB displays excellent biocompatibility, highlighting its significant potential for clinical translation in the treatment of atherosclerosis [115]. Moreover, the incorporation of ticagrelor and PEGylation into raspberry-like platinum and cerium bimetallic nanostructures (PtCe NRs) has been reported to exhibit SOD- and CAT-like activities. This synergistic mechanism involves the inhibition of foam cells and antiplatelet aggregation, effectively attenuating plaque formation (Fig. 5B). Following a 3-month treatment in ApoE^−/−^ mice fed a HFD, there was notable control observed in the size of atherosclerotic lesions, indicating promising potential as a protective measure against the progression of atherosclerosis [117].

### Diabetic wound healing

Apart from the number of metabolic disorders mentioned above, the resulting multiple complications, such as diabetic wound healing, also pose significant challenges to human health and warrant further investigation due to their persistent difficulty in healing [133–135]. In this section, our focus will be on exploring the potential of different nanozymes in enhancing the wound-healing process in individuals with diabetes. The unfavorable wound microenvironment is the main contributing factor to the impaired healing of diabetic wounds, characterized by elevated levels of glucose [136, 137], reduced oxygen supply [138, 139], and an abundance of ROS [140, 141].

**Fig. 6:**
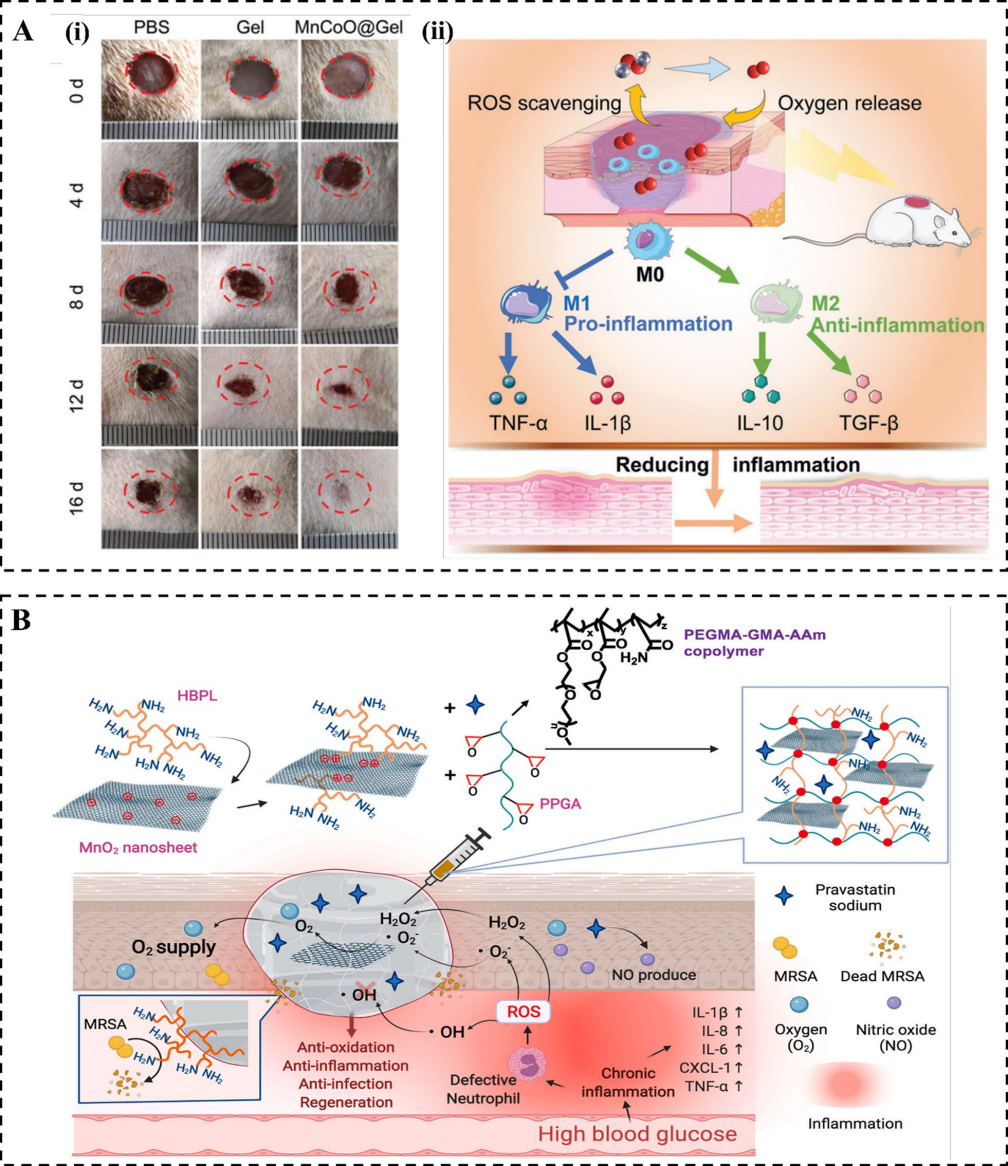
The application of nanozymes in diabetic wound healing. **A** (i) Representative images of the wounds treated with PBS, pure HA hydrogel, and MnCoO@PLE/HA hydrogel for 16 days. (ii) Schematic diagram of MnCoO@PLE/HA hydrogels induce the macrophage polarization direction from M1 to M2 by scavenging ROS and generating oxygen, contributing to the resolution of the excessive inflammation in diabetic wounds. Reproduced with permission [113]. Copyright 2022, Wiley–VCH GmbH. **B** Promoting the healing of infected diabetic wounds by an anti-bacterial and nano-enzyme-containing hydrogel with inflammation-suppressing, ROS-scavenging, oxygen, and nitric oxide-generating properties. Reproduced with permission [118]. Copyright 2022, Elsevier

Recently, the use of nanozyme-based therapeutic systems for diabetic wound treatment has garnered considerable attention due to the combination of nanomaterials and natural enzymes, which offer distinct advantages [142, 143]. For instance, the application of a hydrogel reinforced with a nanozyme MnCoO@PLE has shown promising potential in effectively scavenging endogenous ROS and generating oxygen. The application of this hydrogel has demonstrated remarkable effectiveness in enhancing the healing process of diabetic wounds with oxidative stress. It achieves this through proficiently facilitating the transition of macrophages from an M1 to an M2 phenotype, mitigating inflammation, stimulating cellular proliferation, promoting re-epithelialization, contributing to collagen formation, and fostering angiogenesis (Fig. 6A) [113]. Similarly, Tu et al. found that hyperbranched poly-L-lysine-modified MnO_2_ nanozymes effectively scavenged ROS, generated oxygen, killed bacteria, and protected cells against oxidative stress. Moreover, in vivo studies exhibited that the application of this hydrogel resulted in a reduction of ROS levels, alleviation of inflammation, decreased neutrophil infiltration, enhanced polarization of M2 macrophages, increased secretion of transforming growth factor-β (TGF-β), stimulation of neovascularization, and deposition of thicker collagen. These combined effects effectively facilitated the healing process of infected diabetic wounds (Fig. 6B) [118].

Notably, some nanozymes have been documented to enhance the healing of diabetic wounds by performing multienzyme-like activities. For instance, it was reported that a MoS_2_@Au@BSA NSs nanozyme has the potential to promote diabetic wound healing by exerting GOx-, CAT-, POD-, and SOD-like activities to reduce oxidative stress, alleviate hypoxia, and facilitate glucose oxidation [119]. Both in laboratory experiments and animal studies, it has been observed that the nanozyme hydrogel spray exhibits five different enzyme-like activities, namely SOD-, CAT-, GOx-, POD-, and NOS-like activities. These activities can be activated by the microenvironment of diabetic foot ulcers, resulting in several beneficial effects such as inflammation reduction, alleviation of hypoxia, blood glucose regulation, promotion of angiogenesis, and elimination of pathogenic bacteria. Consequently, this accelerated healing process effectively enhances diabetic wound recovery [54]. PFOB@PLGA@Pt exhibited multiple enzyme activities, including nicotinamide adenine dinucleotide oxidase-, SOD-, CAT-, OXD-, and POD-like activities. It substantially facilitated the healing process of infected wounds in individuals with diabetes [114]. Moreover, Fe_3_O_4_-GOx exhibits inherent GOx-, CAT-, and POD-like activities, enabling it to drive a pH-switchable cascade reaction between glucose and GOx/POD or GOx/CAT. This ability allows for the modulation of the pathological wound microenvironment, thereby aiding in the recovery of impaired healing in diabetic ulcers [120]. Apart from these nanozymes, numerous nanozymes are gradually being explored and unraveled for their role in promoting diabetic wound healing [144–146]. Overall, these investigations suggest that nanozymes play a crucial role in facilitating the healing process of diabetic-infected wounds.

### Others

Diabetic cataracts are a prevalent ocular complication associated with diabetes, often leading to vision loss and blindness among individuals affected by diabetes [147]. The CeO_2_ nanoparticles coated with PEG-PLGA have shown promising results in their ability to function as an efficient scavenger of ROS and as a glycation inhibitor. These characteristics assist in preventing α-crystallin glycation and crosslinking, which in turn helps maintain the transparency of the lens and ultimately reduces the incidence of cataracts in diabetic rats induced with streptozotocin [148]. In addition, CeCl_3_@mSiO_2_ nanoparticles show promise in reducing oxidative stress and inflammation associated with diabetic cataract formation, offering potential therapeutic benefits for alleviating its development and progression [149].

## How to improve the performance of nanozymes by external forces?

Improving the performance of nanozyme by external forces can be achieved through various methods, including light, ultrasound, pH, magnetic fields, and temperature. In this part, the strategies that can help enhance nanozyme performance will be discussed.

### Light

**Fig. 7:**
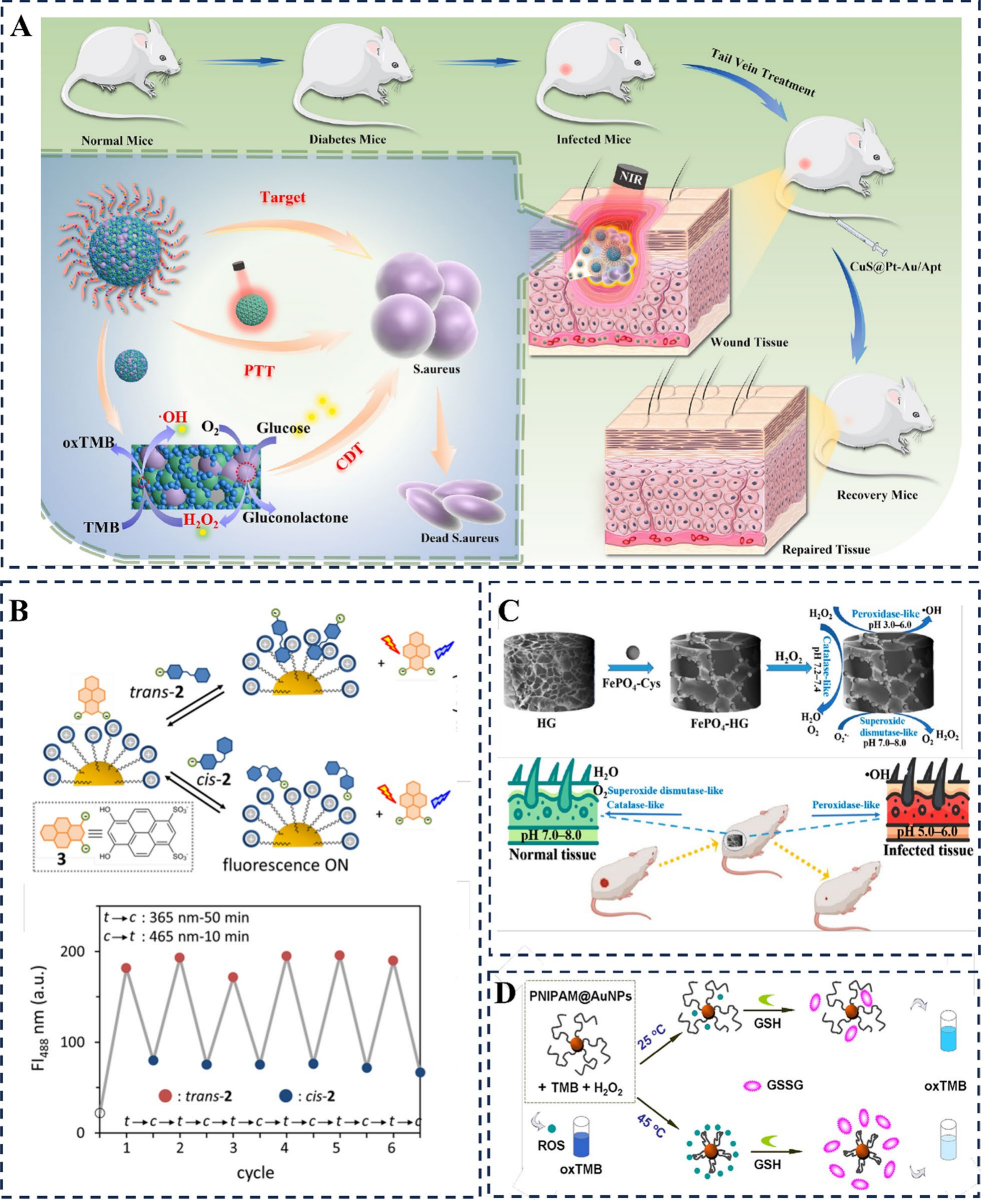
The external forces modulate the performance of nanozymes. **A** Adaptor functionalized nanomaterials can protect nucleic acid aptamers from the degradation of ribozymes and accelerate CuS@Pt-Au/Apt nanoparticles bind onto bacterial cells through aptamers recognition, and the overheat generated by CuS NPs under near-infrared irradiation corrupts bacterial membranes and boosts the release of reactive oxygen species originated from cascade nanozymes with the enhanced and collaborative antibacterial effects [65]. Copyright 2023, Elsevier. **B** Ultraviolet light-induced cis–trans isomerization of a small molecule binding to Au NPs to activate the multi-enzyme-like activities. Reproduced with permission [155]. Copyright 2017, American Chemical Society. **C** FePO_4_ − HG is synthesized and its multifunctional properties as a catalyst with enzyme-like activities are investigated for the treatment of wound infections at different pH conditions. Reproduced with permission [159]. Copyright 2022, American Chemical Society. **D** Thermal stability of POD-mimicking activity of PN-Ceria. Reproduced with permission [160]. Copyright 2015, Elsevier

A new and emerging approach to modulate enzyme-like activity is through light stimulation, which offers exceptional adjustability and precise control over spatial activation [150, 151]. Light-activated nanozyme, also known as photocatalytic nanozyme, are a specific type of nanomaterial that exhibit enzyme-like catalytic activity and can be activated by light. These nanozymes typically consist of inorganic materials, such as metal nanoparticles or semiconductors, that possess photocatalytic properties [65, 152]. The light-responsive behavior of these nanozymes is typically achieved by incorporating light-sensitive molecules or materials into their structure. For example, photosensitive organic molecules or semiconducting nanoparticles can be integrated into the nanozyme system [153, 154]. When exposed to light, these light-responsive components undergo specific photochemical or photophysical processes that trigger changes in the nanozyme’s catalytic activity. Based on the specific wavelength of light that selectively triggers their catalytic activity, light-responsive nanozymes can be categorized into three groups: (1) nanozymes activated by photoisomerization induced by ultraviolet (UV) light; (2) nanozymes stimulated by near-infrared (NIR) irradiation; and (3) nanozymes modulated by visible light. For example, the catalytic activity of gold nanoparticles can be enhanced by incorporating a light-sensitive cofactor, which enables them to be activated in a controlled manner through trans–cis isomerization induced by UV light (Fig. 7B) [155]. Recently, metallic nanostructures including Au, Cu, Pd, and Pt have been found to demonstrate enhanced enzyme-like activity through surface plasmon resonance when excited within the visible to NIR range [156–158]. Interestingly, Yan et al. discovered that the combination of laser irradiation (808 nm) with _PA_Au BPs treatment effectively controlled the immune microenvironment of adipose tissue in mice with obesity [66]. CuS@Pt-Au/Apt NPs, a nano-antibacterial material functionalized with aptamers and responsive to NIR (808 nm), based on cascaded nanozymes with OXD-POD and POD-like activity, has proven successful for the purpose of synergistic therapy aimed at treating wound infections in diabetic mice (Fig. 7A) [65].

### Ultrasound

Ultrasound can enhance the catalytic activity of nanozymes by several mechanisms [161, 162]. Firstly, it enhances the transport of reactants to the nanozyme's surface, thereby facilitating more efficient catalysis [163]. Ultrasound can create acoustic cavitation, which generates microbubbles that collapse and create localized high temperatures and pressures[164]. This phenomenon can enhance the diffusion and penetration of reactants into the nanozyme, leading to improved catalytic activity.

Additionally, ultrasound can also induce physical and chemical changes in the nanozyme structure, leading to modifications in its catalytic properties. For example, ultrasound has the capability to induce the creation of defects or active sites on the nanozyme surface, thereby heightening its catalytic activity [165]. Ultrasound can also modify the morphology and size of the nanozyme, which can affect its catalytic performance [166]. Interestingly, Sun et al. found that the modification of organic sonosensitizer meso-tetra (4-carboxyphenyl) porphine onto Pd@Pt effectively blocked the CAT activity of Pd@Pt, but under ultrasound irradiation, the nanozyme activity was restored to catalyze the decomposition of endogenous H_2_O_2_ into O_2_ [167]. The developed nanozyme system, which can be switched on and off by ultrasound, offers a promising approach to enhance the sonodynamic eradication of healthy tissues. Overall, ultrasound can be used as a tool to improve the performance of nanozymes by improving mass transport, inducing structural changes, and modifying the catalytic properties of the nanomaterial. This can have significant implications for various applications where nanozymes are used, such as biosensing, drug delivery, and metabolic disease modulation.

### pH

The catalytic activity of nanozymes is notably influenced by the pH of the surrounding environment, as it directly affects the ionization status of the functional groups located on their surface [168]. Research suggests that nanozymes can display diverse enzyme activities depending on the pH conditions. For instance, Liao et al. constructed a nanozyme-hydrogel composed of iron phosphate (FePO_4_-HG) that possessed characteristics such as positive charge and macropores and found that FePO_4_-HG exhibits POD-like activity in acidic bacterial infections and shows synergistic effects similar to SOD-CAT in neutral or weak alkaline conditions, thereby improving normal tissues from damage caused by POD-like reactions triggered by exogenous H_2_O_2_ (Fig. 7C) [159]. Similarly, by modulating the pH, Asati et al. demonstrated that nanoceria can be tuned to facilitate the oxidation of the nonfluorescent substrate ampliflu. This oxidation can lead to the formation of either the highly stable fluorescent product resorufin at pH 7.0 or the nonfluorescent resazurin at pH 4.0 [169]. Additionally, a recent research paper presents the advancements in Co-doped mesoporous cerium oxide (Co-m-ceria), which exhibits superior performance at approximately neutral pH levels and exhibits a POD-like catalytic efficiency that surpasses that of pristine m-ceria by 600-fold [170]. Overall, these findings indicate that the activity of nanozymes can be influenced by altering the pH levels in their environment. By observing changes in nanozyme activity at different pH conditions, researchers were able to determine that pH plays a role in modulating its activity. This information can be valuable in understanding the function and regulation of nanozyme, as well as potentially developing strategies to control its activity for various applications.

### Magnetic fields

The regulation of the catalytic activity of nanozymes is of utmost importance in the context of their biomedical applications. Many nanomaterials possess magnetic (such as Fe_3_O_4_) and optical properties, which provide feasibility for developing stimulus-responsive nanozymes [171, 172]. The application of magnetic fields, as an external stimulus, offers distinct benefits in modulating the activity of nanozymes. The magnetic field, characterized by excellent biosecurity and adjustable nature, enables clinical translation and offers the advantage of deep tissue penetration, making it more favorable for biomedical applications. In a recent study, conducted by He et al., they developed a range of iron oxide nanoparticles (IONPs) with varying magneto-thermal conversion capabilities and then systematically investigated the impact of magnetic field stimulation on the peroxidase-like activity of these IONPs. They showed that by applying in situ alternating magnetic field stimulation, the POD-like activity is notably increased without generating any rise in the temperature of the solution. The extent of this activity enhancement is directly linked to the magnetic heating ability of the IONPs, thereby confirming that the local magnetothermal effect primarily contributes to the enhancement of activity [173]. This study revealed that the activity of nanozyme solutions can be controlled by alternating magnetic fields, and there is a positive correlation between the enhancement of enzyme activity and the magnetic heat conversion efficiency of the material itself. This provides important theoretical support for further designing highly magnetic responsive nanozymes to remotely regulate their catalytic activity.

### Temperature

Temperature plays a crucial role in the functionality of natural enzymes, and the enzyme activity changes obviously under different temperature conditions. In contrast to natural enzymes, nanozymes exhibit greater stability in terms of their activity over a wider temperature range. For instance, Wang et al. discovered that PNzyme/MnO_2_ demonstrated stable SOD and CAT mimicking activities between 4 and 80 °C, unlike natural enzymes [174]. Likewise, the POD-mimicking activity of PN-Ceria was examined across different temperatures, spanning from 4 to 60 °C. The results showed relatively consistent activity levels, with the peak catalytic activity observed at 35 °C [160]. Based on the aforementioned partial nanozyme, the temperature has a minor impact on its enzymatic activity. However, existing research indicates that some nanozymes are still affected by temperature variations in terms of their catalytic activity. In particular, the study of thermo-responsive nanozymes provides new ideas and insights for the research on temperature regulation of nanozyme activity. For example, Cheng et al. utilized thermo-responsive poly(N-isopropyl acrylamide) (PNIPAM) as a stabilizer and reducing agent to create a straightforward and efficient approach for regulating the POD-like catalytic activity of PNIPAM@AuNPs in the TMB-H_2_O_2_ oxidation system by manipulating the ambient temperature (Fig. 7D) [175]. Therefore, it is important to consider and optimize the temperature conditions for nanozyme-based applications to achieve their maximum catalytic performance.

In addition, in in vitro experiments, precise control of the ambient temperature is indeed required to mimic and optimize the catalytic activity of nanozymes. However, the in vivo environment is significantly different from the in vitro experimental conditions, so we need to adopt some strategies to address this issue. First, we need to make it clear that nanozymes are designed and optimized to work under specific physiological conditions, which includes a constant temperature in the human body. Therefore, when we study the catalytic activity of nanozymes in vitro, our goal is to determine an optimal temperature window that allows the nanozymes to exhibit optimal catalytic efficiency in that temperature range. Such studies help us understand the possible operating temperature range of the nanozymes in vivo and provide guidance for further in vivo applications. Second, we can enhance the catalytic activity of nanomaterials at constant body temperature by surface modification. For example, the stability and activity of nanozymes at physiological temperatures can be enhanced by introducing specific ligands or coatings. Such surface modifications can not only improve the catalytic efficiency of the nanozymes, but also enhance their affinity for specific substrates, leading to efficient catalysis at constant body temperature. In addition, we can explore the intrinsic catalytic mechanism of nanozymes to understand how they adjust their catalytic activity under different temperature conditions. This may involve the study of the electronic structure and coordination environment of the metal active centers on the surface of the nanozymes, as well as the analysis of the changes in kinetic parameters of the nanozymes at different temperatures. Finally, we can predict and model the behavior of nanozymes in an in vivo environment by means of bioinformatics and computational chemistry. With these computational models, we can predict the catalytic activity of nanozymes at physiological temperatures and provide a theoretical basis for experimental design. In summary, although there are differences in temperature control between in vitro experiments and in vivo environments, with the above strategies, we can optimize the catalytic activity of nanoenzymes and ensure their effectiveness in biomedical applications while maintaining a constant body temperature in vivo.

## The pharmacokinetic characteristics of nanozymes

**Fig. 8:**
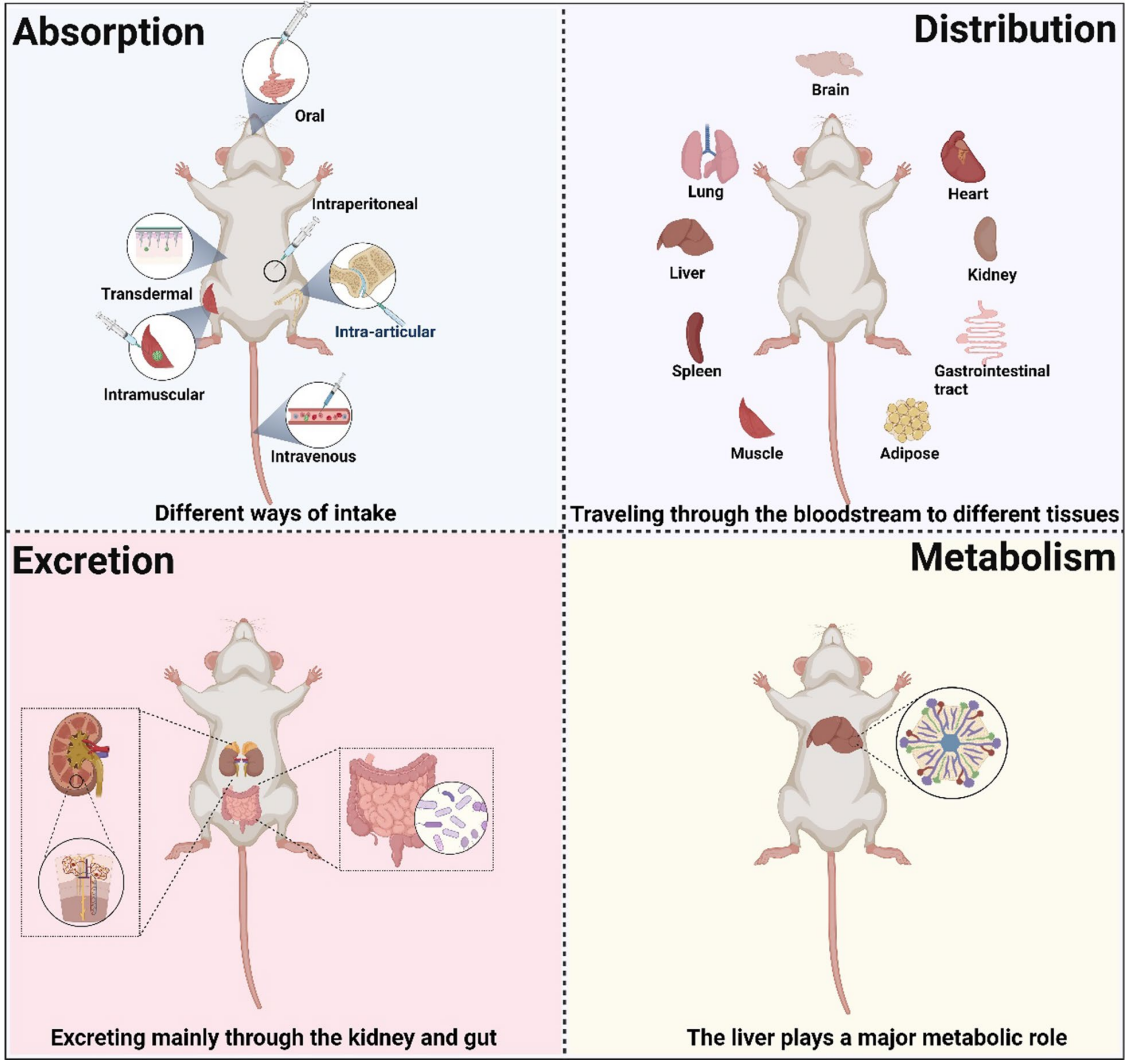
The schematic diagram illustrating the processes of absorption, distribution, metabolism, and excretion of nanozymes in the body

Nanozymes, being nanoscale materials, can undergo a series of physiological processes within the body, including absorption, distribution, metabolism, and excretion. Here, we will give a brief overview of each of these processes (Fig. 8).

### Absorption

Nanozymes have the potential to enter the body through various routes, such as oral ingestion, inhalation, injection, or topical application [176–179]. The absorption of nanozymes is determined by their physicochemical characteristics, such as surface charge, size, and surface functionalization, as these factors affect their interaction with biological barriers like cell membranes or mucosal surfaces [180, 181]. Nanozyme can be internalized by cells through various mechanisms. One common mechanism is endocytosis, where the nanozyme is engulfed by the cell membrane and enclosed in a vesicle [182]. This process can occur through different pathways, such as clathrin-mediated endocytosis [183], caveolae-mediated endocytosis [184], or micropinocytosis [185], depending on the characteristics of the nanozymes and the cell type. Overall, the absorption of nanozyme in cells involves their internalization through endocytosis and subsequent localization in different cellular compartments, where they can exert their catalytic functions. Further research is ongoing to optimize the design and properties of nanozymes for efficient cellular absorption and targeted therapeutic applications.

### Distribution

After absorption, nanozymes can be systematically distributed throughout the body via either the bloodstream or the lymphatic system. Their distribution can be influenced by factors like blood flow, tissue permeability, and interactions with proteins or cells in the circulation [186]. Nanozymes can accumulate in specific tissues or organs, depending on their targeting strategies or the physiological characteristics of the target site [61, 86]. Once inside the cell, nanozymes can be localized in different cellular compartments, such as endosomes, lysosomes, or cytoplasm, depending on the specific targeting or delivery strategy employed [104]. Moreover, the distribution of nanozyme is affected by conditioning, protein corona formation, mononuclear phagocytic system uptake, enhanced permeability and retention effects, targeted therapy, and lymphatic [186]. Protein conditioning in the bloodstream is the primary factor influencing the distribution of nanozymes. Larger nanoparticles are more easily detected and eliminated by opsonins, leading to reduced distribution [187]. Additionally, protein conditioning and physical permeability are crucial for the distribution of nanoparticles in the body, and both factors are influenced by nanoparticle size [188]. Moreover, the hydrophilicity and hydrophobicity of nanoparticles can impact protein conditioning, while intelligent nanoparticles can enhance targeting capability [189]. Therefore, controlling the size and hydrophobicity of nanoparticles and employing intelligent nanoparticle designs can regulate their transmembrane ability, enabling rapid targeted distribution and addressing specific disease treatment requirements.

### Metabolism

Nanozymes are synthetic nanomaterials that mimic the catalytic activity of enzymes, but they do not undergo metabolic transformations or participate in the body’s metabolic processes. Nanozymes are designed to catalyze specific reactions in a controlled manner, but they do not possess the ability to perform the complex metabolic processes that occur in living organisms. Their main function is to enhance or imitate the functionality of natural enzymes for various applications, such as biomedical diagnostics, therapeutics, and environmental remediation.

### Excretion

Nanozymes and their metabolites can be eliminated from the body through various routes, including renal excretion (via urine), biliary excretion (via feces), or exhalation (via breath). The excretion of nanozymes depends on their physicochemical properties, such as surface coating, surface charge, shape, and size. These properties can influence their clearance from the body. Nanozymes that are small enough can be filtered by the kidneys and eliminated through urine [190]. The size cutoff for renal excretion is typically around 5–10 nm, depending on the specific characteristics of the nanozymes and the filtration capacity of the kidneys [191]. Larger nanozymes that are not efficiently cleared by the kidneys may be taken up by liver cells and excreted into the bile. From there, they can be eliminated through feces. Besides, nanozymes can be recognized and engulfed by immune cells in the reticuloendothelial system, such as macrophages. These cells can then transport the nanozymes to the liver or spleen for further processing and elimination. Some nanozymes may be captured by lymphatic vessels and transported to lymph nodes, where they can be degraded and eliminated.

It is crucial to note that the absorption, distribution, metabolism, and excretion of nanozymes can vary depending on their specific properties, administration route, and intended application. Understanding these processes is crucial for assessing the safety, efficacy, and potential therapeutic uses of nanozymes in the field of biomedical research.

## The safety analysis of nanozyme

Nanozymes, which exhibit enzyme-like catalytic activity, have garnered considerable interest in recent times for their potential applications across diverse areas, such as biomedicine, environmental remediation, and energy conversion [192]. However, the safety of nanozymes is a topic of concern and ongoing research.

### Physicochemical properties of nanozyme that influence toxicity

The toxicity mechanisms of nanozymes are dictated by their absorption, translocation, and accumulation within living tissues, which are impacted by the physical and chemical attributes of the nanozymes [193]. Having a comprehensive understanding of these physicochemical properties is crucial for the design and fabrication of nanozymes with enhanced safety characteristics. The toxic potential of nanozymes is contingent upon their physicochemical characteristics, encompassing surface chemistry, shape, size, and chemical composition.

The size of nanozymes is an important factor in determining how they are internalized by cells, which in turn affects their distribution within live tissues and the mechanisms involved. Nanozymes of smaller dimensions possess the capability to traverse cell membranes via translocation, while larger nanozymes employ alternative transport mechanisms such as phagocytosis, micropinocytosis, and non-specific translocation to gain entry into cells [194]. Furthermore, nanozymes exhibit diverse shapes like ellipsoids, cubes, rods, sheets, spheres, and cylinders. When nanozymes possess similar sizes and compositions, their specific shape has the potential to modify their biological activities, such as cellular uptake, biodistribution, deposition, and clearance [195].

Furthermore, surface chemistry plays a pivotal role in exerting a significant influence on toxicity. The surface charge of nanozymes is closely associated with their toxicity properties, influencing their pharmacokinetics and interactions with organelles and biomolecules. Zeta potential has been widely used to assess the surface charge of nanozymes and predict their potential toxicity. Numerous studies have provided evidence that nanoparticles with a positive zeta potential are more inclined to elicit toxic effects in contrast to nanoparticles with a negative zeta potential [196, 197]. Furthermore, the surface of nanozyme can be altered using various coating materials including PEG, PLGA, polylactic acid, lipids, and other substances. These coatings effectively alter the physicochemical properties of the nanozyme [31, 198, 199]. Modifications made to the surface of nanozymes have a significant impact on their pharmacokinetics, altering their clearance, elimination, and biodistribution processes. These factors are intricately connected to the toxicological impact of nanozymes within the organism.

### The mechanisms of toxicity

The toxic potential of nanoparticles is influenced by their physicochemical properties. To evaluate nanoparticle toxicity, a variety of in vitro and in vivo models have been employed, such as cell lines, 3D cell cultures, rodents, zebrafish, and other experimental systems [200–202]. The precise mechanism of nanomaterials toxicity is not yet fully understood. However, it is known that multiple mechanisms are involved, including mitochondrial dysfunction, DNA damage, and other related processes. Despite continuous research efforts, a comprehensive understanding of the toxicity of nanoparticles remains elusive, as no singular mechanism or signaling pathway has been definitively identified [203]. Furthermore, the intricate interplay and interaction between different signaling pathways further adds complexity to our comprehension of the exact mechanism underlying nanoparticle toxicity. Therefore, the investigation of the mechanism of nanozyme toxicity is a heavy and long-term task, which deserves further exploration.

### The assessment approach of toxicity

The assessment approach of toxicity for nanozymes involves several steps and methods to determine the potential adverse effects of these nanomaterials on living organisms. We gathered published literature to compile a brief overview of the currently available measures for evaluating the toxicity of nanomaterials. In vitro cellular experiments are commonly employed to assess the toxicity of nanoparticles due to their low cost and simplicity, allowing for deeper investigation into the underlying molecular mechanisms [204]. Experiments that can be used to determine toxicity based on in vitro cell experiments include cell viability, apoptosis, mitochondrial dysfunction, oxidative stress, and genotoxicity [190, 205]. Furthermore, in vivo animal studies provide the advantage of assessing the toxicological impact of nanomaterials within intricate physiological environments [206]. Recently, conventional toxicology studies have predominantly relied on parameters such as body weight, organ tissue weight, pathological damage in significant organ tissues, and blood biochemical indices [104, 105]. However, these approaches are inadequate in providing a complete understanding of nanozyme toxicity. With the rapid development of omics technologies, which offer a comprehensive molecular profiling approach for screening nanoparticle toxicity at low exposure levels, identifying novel biomarkers and targets of nanotoxicity both in vivo and in vitro, and detecting minuscule variations within a cell compared to classical methods.

To ensure the safety of nanozymes, it is crucial to conduct comprehensive toxicity assessments, including in vitro and in vivo investigations. Researchers need to evaluate the potential adverse effects of nanozymes on various biological systems, such as cells, tissues, and organisms. Additionally, the design and engineering of nanozymes should consider factors that minimize their potential toxicity, such as surface modifications and the use of biocompatible materials. In conclusion, while nanozymes exhibit significant promise in diverse applications, their safety needs to be thoroughly investigated. Further research is required to comprehend the toxicity mechanisms and develop safe and effective nanozymes for practical use.

## The advantages and challenges of nanozyme

### The advantage of nanozyme

Nanozyme refers to nanomaterials that possess enzyme-like activities. They have garnered substantial interest in recent years owing to their distinctive characteristics and promising applications. These nanozymes can be tailored to replicate the functionalities of specific natural enzymes engaged in metabolic pathways.

### An alternative to existing medicine

These nanozymes imitate the functionalities of natural enzymes and present numerous benefits compared to conventional drugs. For example, the treatment of diabetic foot ulcers requires addressing multiple aspects such as inflammation reduction, hypoxia relief, blood glucose control, angiogenesis promotion, and elimination of pathogenic bacteria [207]. However, the effectiveness of therapy is significantly hindered by the complex interactions and interdependencies among different drugs and the unique microenvironment of diabetic foot ulcers. According to the findings of Shang et al., the ultrasound-enhanced hydrogel spray, functioning as a multi-enzyme-like nanozyme, responds to the microenvironment of diabetic foot ulcers and efficiently expedites the healing process of diabetic wounds by mitigating inflammation, relieving hypoxia, lowering blood glucose levels, promoting angiogenesis, and eliminating pathogenic bacteria [54]. This study emphasizes the use of multienzyme-like nanozymes as a competitive strategy in the development of comprehensive therapies for diabetic foot ulcers. Moreover, GOx nanozymes can catalyze the oxidation of glucose, providing a basis for glucose sensing and monitoring in diabetic patients. This enables real-time glucose monitoring without the need for invasive blood sampling, offering a more convenient and painless alternative for diabetes management [208].

### Improve the targeting and bioavailability of medicine

Nanozymes can be utilized for precise drug delivery by incorporating specific ligands onto their surface, enabling them to selectively bind to disease-related biomarkers. This targeted approach facilitates the direct delivery of therapeutic agents to the affected tissues and minimizes off-target effects and enhances the efficacy of drug treatments for metabolic diseases. For example, a new strategy for noninvasive active-targeting therapy for ischemic stroke involves utilizing a mesoporous Prussian blue nanozyme coated with a neutrophil-like cell membrane. This innovative approach enhances the targeted delivery of therapeutic agents to the injured brain by capitalizing on the inherent relationship between inflamed brain microvascular endothelial cells and neutrophils following a stroke [209]. In order to enhance biocompatibility and facilitate cellular uptake, Pt nanozymes were coated with triphenylphosphonium-conjugated liposomes, enabling them to evade the lysosomal barrier, penetrate the cell membrane, and specifically target mitochondria for efficient scavenging of mitochondrial O_2_^•−^ and alleviation of hypoxia [210].

### Reduce the adverse effects of medicine

Nanozymes have the potential to decrease the adverse effects of medicines by improving their specificity and minimizing off-target effects. Nanozymes can encapsulate drugs, protecting them from degradation and improving their stability. This allows for better control over drug release, ensuring sustained and controlled delivery. Additionally, nanozymes can enhance the solubility of limited water-soluble drugs, enhancing their bioavailability. Nanozymes can be designed to react to particular cues, including pH variations, temperature changes, or specific biomarkers. This enables controlled drug release at the desired site or under specific conditions. Overall, nanozymes have great promise in improving the targeting and bioavailability of medicines. Their unique properties and versatility make them invaluable assets in the advancement of sophisticated drug delivery systems for diverse medical conditions.

### The challenge of nanozyme

The advantages of nanozymes make them highly promising for a diverse array of applications, offering improved performance, cost-effectiveness, and stability compared to natural enzymes. However, research on how nanozymes modulate metabolic disease is currently at the tip of the iceberg, leaving numerous scientific and technological challenges that need to be thoroughly investigated and resolved.

### The mechanism of action deserves further study

Although nanozymes have shown remarkable therapeutic efficacy in established animal models, the intrinsic biological mechanisms utilized by these nanozymes still remain elusive. Additionally, it is imperative to investigate the metabolic pathway of nanozymes within the bloodstream. Further exploration of the in vivo behavior of nanozymes is of utmost significance, necessitating a more comprehensive understanding. The absorption, distribution, metabolism, excretion, and bioavailability of nanozymes need to be investigated.

### Biocompatibility and safety concerns

Despite their immense potential, there are still challenges to overcome in the development and clinical translation of nanozymes. These include ensuring biocompatibility, optimizing catalytic efficiency, and addressing potential toxicity concerns. The biosafety of nanozymes poses an unavoidable challenge prior to their translation into clinical applications. Unlike natural enzymes, the metabolic fate of nanozymes is a critical concern that demands attention. In certain instances, the metal ions employed in nanozyme synthesis may not be essential for the functioning of organisms, while certain components within nanozymes may possess potential toxicity, potentially compromising their therapeutic efficacy. Considering genotoxicity, the small size and high surface activity of nanozymes facilitate their penetration across cell membranes, enabling direct or indirect interaction with cellular genetic material. Especially for nanozyme carrying metal ions, they can induce chromosome or DNA breaks through mechanisms such as oxidative stress or inflammation after entering cells. Limited research articles have investigated the potentially toxic effects of nanozymes, including genotoxicity and reproductive toxicity. In summary, a thorough exploration of the metabolism and biosafety of nanozymes is crucial for advancing them to the clinical trial stage.

### Reproducibility and scalability limitations

While nanozymes offer great potential for various applications, there are certain limitations related to their reproducibility and scalability that need to be addressed. One challenge is ensuring the reproducibility of nanozyme synthesis and characterization. Various factors, including the method of synthesis, reaction parameters, and surface alterations, can have an impact on the properties and catalytic activity of nanozymes. Small variations in these parameters can lead to significant differences in the performance of nanozymes. Therefore, it is crucial to establish standardized protocols and characterization techniques to ensure the reproducibility of nanozyme synthesis and evaluation. Another limitation is the scalability of nanozyme production. Many nanozymes are synthesized using complex and time-consuming methods, which may not be easily scalable for large-scale production [73]. Additionally, the synthesis of some nanozymes requires the use of expensive or toxic reagents, further hindering their scalability.

### Regulatory considerations

Nanozymes are a relatively new and emerging technology, and there may be a lack of specific regulations tailored to their unique characteristics and applications. Existing regulatory frameworks may not have provisions that directly address nanozymes, leading to uncertainties and challenges in navigating the regulatory landscape [211]. Regulatory requirements for nanozymes may differ across countries and regions, leading to challenges in international trade and collaboration [212, 213]. Achieving harmonization and alignment of regulatory frameworks worldwide can facilitate the global development and use of nanozymes. International cooperation and standardization efforts are necessary to address this challenge.

Nanozymes offer several advantages in the treatment of metabolic diseases. Their high catalytic activity enables efficient and specific detection of disease-associated metabolites. In addition, nanozymes can be tailored for targeted delivery to specific tissues or cells affected by metabolic disorders, thereby enhancing therapeutic efficacy. In addition, their stability ensures prolonged activity and storage, making them feasible for clinical application. However, there are challenges in using nanozymes for the treatment of metabolic disorders. Ensuring biocompatibility to prevent adverse reactions, scaling up production for widespread use, and meeting regulatory standards for clinical approval are significant hurdles. By carefully considering the advantages and disadvantages of nanozymes in metabolic diseases, researchers can better assess their potential to improve diagnostic and therapeutic strategies in this area.

## Conclusion and future perspectives

In conclusion, the treatment of metabolic diseases, including obesity, diabetes, and cardiovascular disease, as well as diabetic wounds, remains a challenging task in clinical practice, with current approaches yielding limited therapeutic effects. Nanozymes, with their enzyme-like activities, stability, and targeted delivery capabilities, have proven to be a highly promising tool in the field of metabolic disease management, serving as efficient catalysts for biochemical reactions. These outcomes are achieved by modulation of glucose uptake, facilitation of glycogen synthesis, increased insulin sensitivity and reduced insulin resistance, anti-inflammation and anti-oxidation, suppression of infection, and deeper tissue protection. Despite the growing body of research on nanozymes, the process of translating them into clinical applications has faced significant obstacles, resulting in only a limited number of nanozymes being approved and brought to market. To accelerate the application of nanozymes in the market, future research can focus on the following points:

- Safety is the cornerstone of efficacy. Studies assessing the effects of nanozymes should incorporate more rigorous evaluations of their efficacy and safety, which can greatly aid in the process of selecting the most effective nanozymes for subsequent clinical research.
- In-depth analysis of the mechanism of action. It is crucial to conduct extensive mechanistic investigations, including the elucidation of intracellular signaling pathways and understanding the interaction between antioxidant nanozymes and the in vivo environment. Moreover, the bioavailability of functional nanozymes and their effects on whole-body metabolism should be researched.
- New materials are the source of vitality. Without a doubt, the future holds the promise of more novel nanozyme being developed. Through innovative designs, the use of new materials, and the loading of diverse drugs, nanozyme will continue to be extensively explored in the management of general homeostasis. This, in turn, will propel clinical research, mass production, and eventual clinical application.
- Interdisciplinary collaboration drives research. The research of nanozymes should involve interdisciplinary collaboration in chemistry, materials science, medicine, biology, computer science, optics, and electronics to advance human life and health management.
- Regulations correct the path for market application. Regulations play a role in correcting the path for the market application of nanozymes by establishing guidelines, standards, and oversight to ensure their safe and effective use. This may involve assessing their potential risks, setting quality control measures, and determining appropriate use cases for these innovative products.

As the field of nanozyme advances, it holds the potential to revolutionize metabolic disease management, providing patients with effective and tailored therapeutic approaches for improved metabolic disturbance outcomes. The constant evolution of nanozyme technology and as additional investigations are conducted to address the aforementioned challenges associated with nanozymes will undoubtedly pave the way for cutting-edge advancements and transformative breakthroughs as promising candidates for modulating metabolic disease and overall well-being.

## Data Availability

Not applicable.

## Abbreviations

ACLY: ATP citrate lyase
Akt: Protein kinase B
AMPK: Adenosine 5'-monophosphate (AMP)-activated protein kinase
ATP: Adenosine triphosphate
ATS: Adipocyte-targeting sequence
ASGPR: Asialoglycoprotein receptor
CAT: Catalase
FA: Folic acid
GNP: Gas-generating nanoparticles
Gox: Glucose oxidase
GS: Glycogen synthase
HFD: High-fat diet
LDL: Low-density lipoprotein
mTOR: Mammalian target of Rapamycin
NADH: Nicotinamide adenine dinucleotide
NOS: Nitric oxide synthase
NPs: Nanoparticles
OXD: Oxidase
PDA: Polydopamine
pGSK3β: Phospho-glycogen synthase kinase3β
PI3K: Phosphoinositide 3-kinase
PLG: Poly (D, L-lactide-co-glycolide)
PLGA: Poly (lactic-co-glycolic acid)
PMPB: Prussian blue
POD: Peroxidase
ROS: Reactive oxygen species
SOD: Superoxide dismutase
STZ: Streptozotocin
T2D: Type 2 diabetes
TNF-α: Tumor necrosis factor-α
WHO: World Health Organization
